# Intracellular trafficking of HIV-1 Gag *via* Syntaxin 6-positive compartments/vesicles: Involvement in tumor necrosis factor secretion

**DOI:** 10.1016/j.jbc.2024.105687

**Published:** 2024-01-26

**Authors:** Naomi Tsurutani, Fumitaka Momose, Keiji Ogawa, Kouichi Sano, Yuko Morikawa

**Affiliations:** 1 Graduate School of Infection Control Sciences, Kitasato University, Tokyo, Japan; 2 Osaka Medical and Pharmaceutical University, Takatsuki, Osaka, Japan

**Keywords:** HIV, gag, SNARE, syntaxin, TNFα, trafficking

## Abstract

HIV-1 Gag protein is synthesized in the cytosol and is transported to the plasma membrane, where viral particle assembly and budding occur. Endosomes are alternative sites of Gag accumulation. However, the intracellular transport pathways and carriers for Gag have not been clarified. We show here that Syntaxin6 (Syx6), a soluble N-ethylmaleimide-sensitive factor attachment protein receptor (SNARE) involved in membrane fusion in post-Golgi networks, is a molecule responsible for Gag trafficking and also for tumor necrosis factor-α (TNFα) secretion and that Gag and TNFα are cotransported *via* Syx6-positive compartments/vesicles. Confocal and live-cell imaging revealed that Gag colocalized and cotrafficked with Syx6, a fraction of which localizes in early and recycling endosomes. Syx6 knockdown reduced HIV-1 particle production, with Gag distributed diffusely throughout the cytoplasm. Coimmunoprecipitation and pulldown show that Gag binds to Syx6, but not its SNARE partners or their assembly complexes, suggesting that Gag preferentially binds free Syx6. The Gag matrix domain and the Syx6 SNARE domain are responsible for the interaction and cotrafficking. In immune cells, Syx6 knockdown/knockout similarly impaired HIV-1 production. Interestingly, HIV-1 infection facilitated TNFα secretion, and this enhancement did not occur in Syx6-depleted cells. Confocal and live-cell imaging revealed that TNFα and Gag partially colocalized and were cotransported *via* Syx6-positive compartments/vesicles. Biochemical analyses indicate that TNFα directly binds the C-terminal domain of Syx6. Altogether, our data provide evidence that both Gag and TNFα make use of Syx6-mediated trafficking machinery and suggest that Gag expression does not inhibit but rather facilitates TNFα secretion in HIV-1 infection.

The Gag protein of HIV, the main structural component of virus particles, is synthesized in the cytosol and assembles into viral particles at the plasma membrane (PM) and/or endosomes. Gag consists of 4 domains, N-terminal matrix (MA), central capsid (CA), nucleocapsid (NC), and C-terminal p6 domains, each of which plays a specific and essential role in particle assembly and budding (1, 2). MA contains a bipartite membrane-binding signal, an N-terminal myristoyl moiety, and a highly basic region (HBR) enriched with positively charged amino acids (residue 15–32). The myristoyl moiety mediates Gag membrane binding through hydrophobic interactions with the membrane, whereas the HBR directs Gag to preferentially bind to the PM through the electrostatic interactions with phosphatidylinositol 4,5-bisphosphate [PI(4,5)P_2_], a PM-specific acidic phospholipid (3, 4). CA is composed of the N- and C-terminal domains, both of which cooperatively form a Gag capsid lattice consisting of hexametric rings of CA (5, 6, 7). NC contains two Zn finger motifs and multiple basic amino acids, which promote the packaging of HIV genomic RNA into viral particles. The NC-RNA interaction facilitates Gag assembly (8, 9), likely by utilizing the RNA as a scaffold for Gag assembly. The p6 domain recruits endosomal sorting complexes required for transport proteins, such as TSG101 and AIP-1/ALIX, to the site of particle assembly and facilitates particle budding (10, 11, 12). These findings provide evidence that HIV makes use of the endosomal machinery for particle budding (13).

Unlike positive-strand RNA viruses that are synthesized at the endoplasmic reticulum and Golgi, HIV Gag does not enter the classical secretory pathway but targets the PM and endosomes, suggesting that Gag makes use of alternative trafficking machinery. Electron microscopy has repeatedly shown Gag accumulation and particle production at the PM, suggesting that the PM is the primary site of Gag assembly. However, multiple studies have also observed intracellular virions within late endosomes (LE)/multivesicular bodies (MVB), especially in macrophages (13, 14). Although these findings suggest that LE is an alternative site of HIV assembly, subsequent studies using membrane-impermeant dyes and ion-abrasion scanning electron microscopy have revealed that the virion-containing compartments connect to the PM (15, 16). This observation implies that the seemingly intracellular virions likely represent sequestered HIV by membrane invagination at the cell surface. Compelling evidence for PM assembly has been provided by recent studies in which Gag was initially targeted to the PM and assembled into particles and inhibition of endocytosis did not impair HIV production (17, 18). These data have suggested that a fraction of Gag can be endocytosed from the PM, resulting in dead-end products. Total internal reflection fluorescent microscopy (TIRFM) and fluorescence resonance energy transfer (FRET) assays have also supported this PM assembly model (17, 19, 20). However, the significance of Gag localization to the LE is still controversial. In fact, inhibition of lysosomal degradation induced Gag accumulation at the LE and significant increase in HIV production (21).

Although the site of Gag assembly has been extensively studied (above), it remains unclear how Gag is transported to the assembly site(s). It is not plausible that newly synthesized Gag molecules passively diffuse and reach the assembly sites. Pulse-chase studies have shown that Gag binds to the membrane soon after Gag synthesis (within 10 min) but assembles into virus particles over several hours (22, 23), suggesting that nascent Gag soon associates with the intracellular membrane and is then transported to the site for particle assembly (24). Alternatively, Gag may be transported to the assembly site quickly and form virus particles slowly. However, the latter is less likely, since cell surface observations by TIRFM and scanning ion conductance microscopy have revealed that Gag particle assembly at the PM is completed rapidly (within 10 min) and released within 30 min (20, 25, 26). Several studies have documented the cellular machinery potentially involved in intracellular trafficking of Gag. They include the *trans*-Golgi network (TGN) and post-Golgi pathways mediated by clathrin adaptors, AP1, AP3, and GGA (27, 28, 29), the TGN-associated E3 ubiquitin ligase POSH (30), lipid rafts (23), and cytoskeletal networks (31, 32). The clathrin adaptors mediate distinct vesicle transport pathways: AP1, between the TGN and endosomes; AP3, the TGN/endosomes to the LE/MVB; GGA, the TGN to endosomes (33, 34). Both AP1 and AP3 interact with Gag MA and depletion or dominant-negative inhibition of these adaptors induced Gag relocation and particle reduction (27, 28). Disruption of ADP ribosylation factor (Arf) function, imposed by GGA overexpression, similarly impaired particle production (29). Because the cargo-adaptor-clathrin complex is a large and complicated carrier, it may possibly pick up Gag during vesicle transport. Intracellular membrane trafficking and fusion are also regulated by the soluble N-ethylmaleimide-sensitive factor attachment protein receptor (SNARE) complex (35). It has been shown that soluble N-ethylmaleimide-sensitive factor (NSF), an ATPase for SNARE recycling, is involved in HIV particle production (36). The majority of SNARE molecules are localized in specific subcellular compartments, but some SNAREs dynamically move between many intracellular compartments (37, 38). Also, lipids themselves can be carriers for protein transport. Lipid transport after synthesis generates an asymmetric distribution of the lipids within the cell (*e.g.*, lipid rafts and phosphatidylinositol phosphates [PIPs]) (39). Lipid rafts are generated at the Golgi and accumulated at the PM. It is well documented that Gag strongly associates with lipid rafts and that disruption of lipid rafts impairs particle production (23, 40). PI(4,5)P_2_ is essential for Gag localization to the PM (41, 42), implying a potentially crucial player in the Gag transport; however, it remains unclear how Gag is recruited to the PM before Gag-PI(4,5)P_2_ interactions. Microtubules and actin networks are utilized for virus entry and egress (31). A previous study has shown that depletion or dominant-negative inhibition of the kinesin KIF4, a motor of microtubule-based transport, induces Gag accumulation at the perinuclear region and reduces particle production (43). However, opposite conclusions have been provided by studies using a microtubule-disrupting drug nocodazole, showing neither reduction in particle production nor Gag relocation (17, 21).

Identification of host factors for viruses often uses protein-binding assays and gene knockout/knockdown technology. We have previously shown that HIV-1 Gag transport to the PM and subsequent particle assembly are reproducible in spheroplasts of yeast *Saccharomyces cerevisiae* (44, 45). Numerous genetic mutants including deletion mutants have been isolated in yeast and are available for the study of cellular factors and machinery (46, 47). We used the genetic mutants with defects in post-Golgi membrane trafficking, to identify Tlg1, a SNARE that resides around the TGN and early endosomes (EE) (48), as a potential host factor for Gag transport. In the present study, we show that in human cells, its orthologue Syntaxin6 (Syx6) binds the Gag MA domain and regulates the transport of Gag-loaded vesicles. Depletion of Syx6 and the defect of the MA-Syx6 interactions reduced particle production, with Gag distributed diffusely in the cytoplasm. These findings suggest that Syx6 is responsible for the intracellular trafficking of Gag. Interestingly, we find that tumor necrosis factor-α (TNFα) also binds Syx6 and is transported *via* Syx6-positive vesicles. The cotransport of TNFα and Gag *via* Syx6-mediated vesicles may partly explain the upregulation of TNFα upon HIV-1 infection.

## Results

### Syx6 and Syx12 regulate Gag transport and viral particle production in human cells

**Figure 1:**
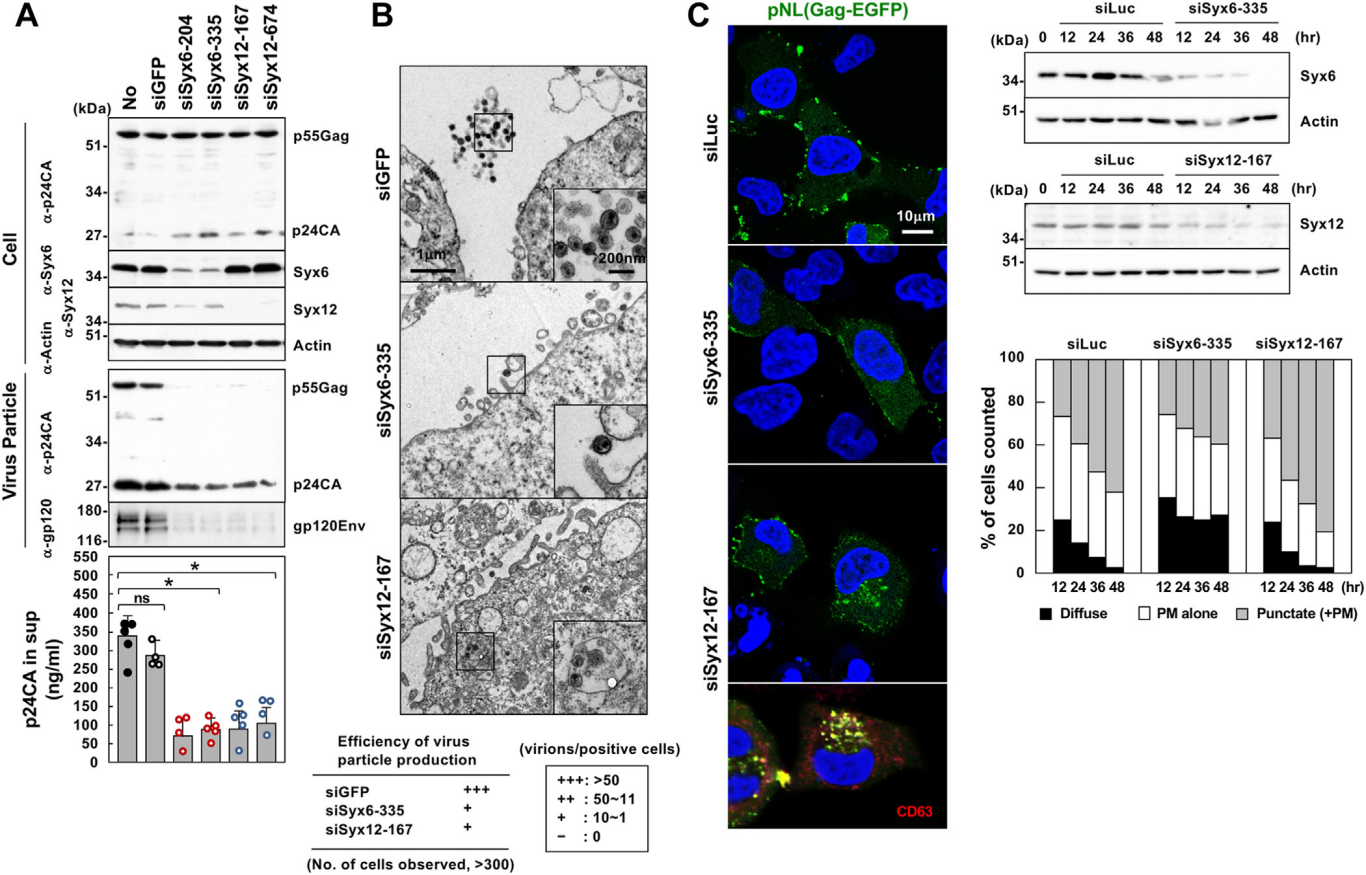
**Knockdown of Syx6 and Syx12 impairs HIV-1 Gag transport to the PM and particle release in human cells.***A* and *B*, HIV-1 particle release. 293T (*A*) and HeLa (*B*) cells in 6-cm dishes were transfected with 80 nM Syx6 siRNAs (siSyx6-204 and siSyx6-335), Syx12 siRNAs (siSyx12-167 and siSyx12-674), or GFP siRNA (siGFP, control) and then with 1 μg of pNL43. *A*, at 48 h posttransfection with pNL43, 293T cells and purified HIV-1 particles were analyzed by western blotting using anti-HIV-1 p24CA, anti-Syx6, anti-Syx12/13, anti-actin mAbs, and anti-HIV-1 gp120Env Ab. Representative blots are shown. Particle yields were quantified by HIV-1 p24CA antigen ELISA. Data are the mean with SD from 4 independent experiments. ∗*p* < 0.05; ns, not significant, Mann–Whiteny *U* test. *B*, HeLa cells were subjected to electron microscopy at 48 h posttransfection. Scale bars: 1 μm (panels); 200 nm (insets). *C*, intracellular localization of HIV-1 Gag. HeLa cells (in 12-well plates) were cotransfected with 80 nM Syx6 siRNA (siSyx6-335), Syx12 siRNA (siSyx12-167), or luciferase siRNA (siLuc, control) and 0.2 μg of a pNL43 derivative expressing Gag-EGFP. The cells were temporally harvested and analyzed by western blotting with anti-Syx6, anti-Syx12/13, and anti-actin mAbs. At several time points, the cells were fixed and subjected to confocal microscopy. Nuclei were stained with DAPI. Representative images in each sample (of 36 h postinfection) were shown at the same magnification. siSyx12-167-transfected cells were also costained with anti-CD63 mAb. For semi-quantification of Gag localization, 100 to 150 Gag-EGFP-positive cells were observed at each time point and the number of cells with each pattern of Gag distribution (diffuse in the cytoplasm, at the PM alone, or cytoplasmic puncta) was counted.

The use of a series of yeast genetic mutants (Table S1) identified Tlg1, a SNARE for transport between the late Golgi and EE, and Pep12, a SNARE for transport from the late Golgi to prevacuolar endosomes/LE, as host factors for HIV-1 Gag particle release (Text S1 and Fig. S1). We explored whether Syx6 and Syx12, human orthologues of Tlg1 and Pep12 were responsible for Gag transport in human cells. Syx6 and Syx12 are SNARE molecules that mediate vesicle fusion in the TGN and endosomes, respectively (49, 50). When 293T cells were cotransfected with HIV-1 proviral clone pNL43 and siRNAs targeting Syx6 (siSyx6) or Syx12 (siSyx12), endogenous Syx6 and Syx12 were specifically reduced to very low levels, while the Gag expression levels were unaffected (Fig. 1*A*). Depletion of the Syx6 and Syx12 significantly reduced the production of HIV-1 particles. Quantification of p24CA antigen in the culture media indicated that Syx knockdown led to approximately 70% reduction in particle yields compared with cells cotransfected with non-cognate siRNA (siGFP) (Fig. 1*A*). A similar level of reduction in particle yields was observed when the *env*-deleted pNL43 derivative was used (Fig. S2*A*), indicating that the reduction was not due to an indirect effect in which a block in the Env transport through the TGN might halt intracellular trafficking of Gag. When 293T cells were cotransfected with pNL43, siSyx6/siSyx12, and the siSyx-resistant Syx6/Syx12 expression plasmids, their knockdown phenotypes (reduction of particle production) were rescued (Fig. S2*B*).

HeLa cells were similarly cotransfected with pNL43 and the siRNAs. Approximately 300 cells in each sample were analyzed by electron microscopy. Compared with HeLa cells cotransfected with siGFP, where mature HIV-1 particles were efficiently released (>50 virions/cell), only very few particles were produced from cells cotransfected with siSyx6 and siSyx12 (1–10 virions/cell) (Fig. 1*B*). In siSyx12-cotransfected cells, vesicles accommodating particles, suggestive of intracellular budding or internalization of particles, were clearly visible in approximately 40% of cells containing HIV-1 particles. This finding was not noted in siGFP- or siSyx6-cotransfected cells. Essentially similar results were obtained in 293T cells (Fig. S3*A*). Together, these results indicate that Syx6 and Syx12 are positive factors responsible for HIV-1 particle production.

For Gag localization, 293T and HeLa cells were transfected with a pNL43 derivative that produces Gag-EGFP and temporally observed by confocal microscopy (Fig. S4). Since Gag-EGFP affects untagged Gag’s behaviors, especially at stages of particle assembly and budding (26, 51), we compared Gag distribution patterns (diffuse in the cytoplasm, at the PM alone, and cytoplasmic puncta) (17, 52) in cells expressing Gag-EGFP alone with cells coexpressing Gag-EGFP and unlabeled Gag. A pNL43 derivative expressing Gag-EGFP was singly transfected or cotransfected with a pNL derivative expressing untagged Gag to HeLa and 293T cells, and Gag-EGFP distribution pattern was temporally observed by confocal microscopy (Fig. S4). In HeLa cells (Fig. S4*A*), Gag accumulation at the PM was frequently observed at early time points (55–60% of Gag-EGFP-positive cells) but cytoplasmic puncta, especially large puncta, became dominant at late time points (up to 65% of Gag-EGFP-positive cells), suggestive of endocytosis. Similar Gag relocation was observed when Gag-EGFP was coexpressed with untagged Gag. In 293T cells (Fig. S4*B*), Gag accumulation at the PM was predominant throughout the period (50–60% of Gag-EGFP-positive cells). Punctate Gag signals were observed but, unlike in HeLa cells, did not significantly increase at late time points. No apparent differences in Gag distribution were observed in cells expressing Gag-EGFP alone and in cells expressing Gag-EGFP plus untagged Gag. However, cell rounding was more prominent in cells coexpressing with untagged Gag. This finding may be correlated with efficient particle production when Gag-GFP was coexpressed with untagged Gag (26, 51).

Then, we examined whether Gag transport pathways were altered in Syx6-and Syx12-depleted cells. HeLa and 293T cells were cotransfected with a pNL43 derivative that produces Gag-EGFP and with siSyx6 or siSyx12 and were temporally observed by confocal microscopy (Figs. 1*C* and S3*B*). In luciferase siRNA (siLuc)-transfected HeLa cells (control), Gag was accumulated at the PM at early time points and cytoplasmic puncta became evident at late time points (Fig. 1*C*). More than 100 Gag-EGFP-positive cells were subjected to analysis of Gag distribution patterns (Fig. 1*C*, right). In Syx6-depleted cells, Gag-EGFP was found in a more diffuse and less punctate cytoplasmic distribution. In contrast, cytoplasmic puncta that partially colocalized with CD63, were dominant in Syx12-depleted cells, particularly at late time points (Fig. 1*C*, left). Similar alternations in Gag localization were observed in 293T cells transfected with siSyx6 and siSyx12. In Syx6-depleted cells, Gag was relatively diffusely distributed in the cytoplasm, whereas in Syx12-depleted cells, cytoplasmic puncta were evident (Fig. S3*B*). These data indicate that depletion of Syx6 and Syx12 altered Gag localization in both HeLa and 293T cells.

### Gag interacts with Syx6 *via* MA-SNARE domain interaction

**Figure 2:**
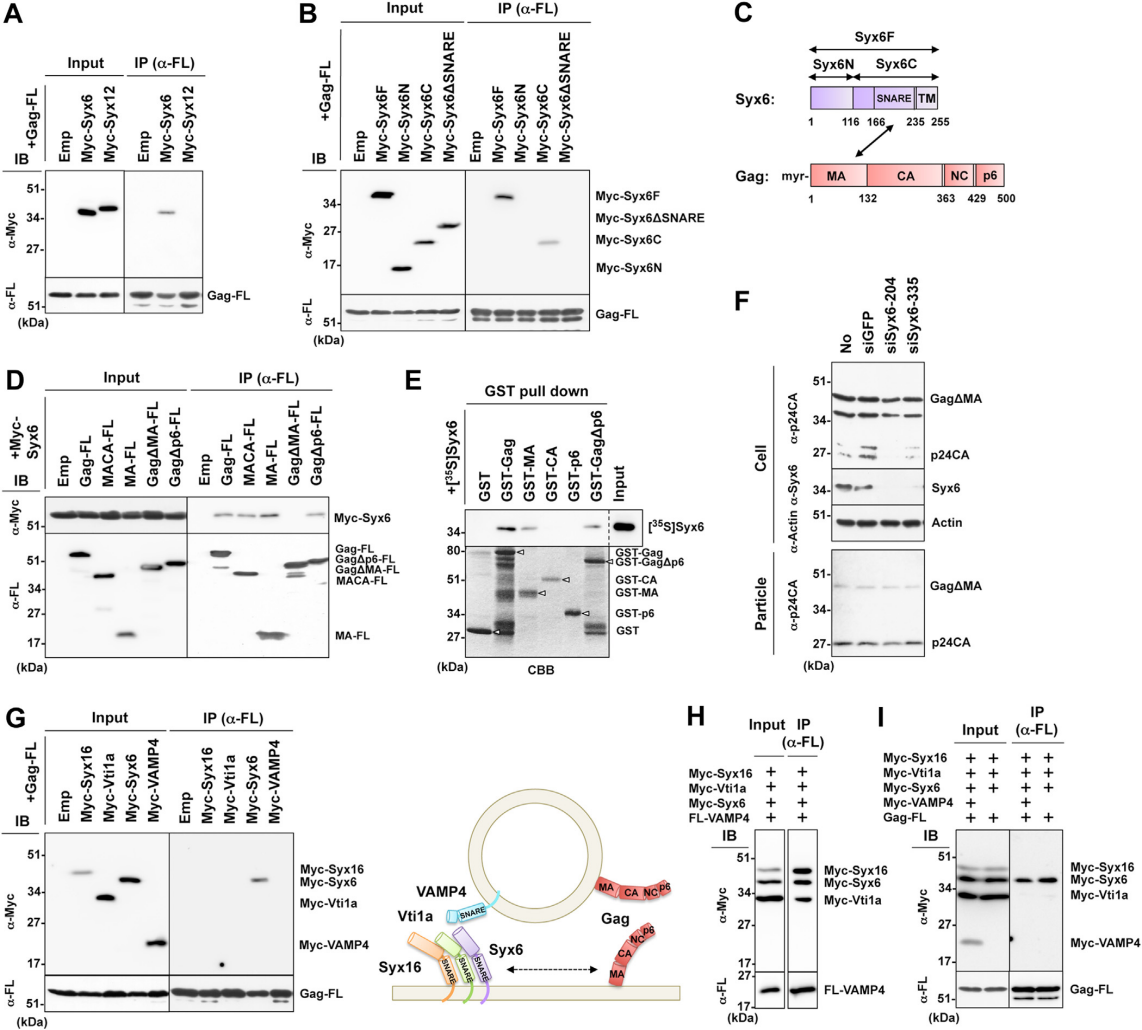
**Syx6 interacts with the Gag MA domain.** Constructs were expressed in 293T cells, lysed in lysis buffer containing 1% Triton-X 100, and subjected to immunoprecipitation with anti-FLAG mAb followed by western blotting with anti-FLAG and anti-Myc mAbs (*A*, *B*, *D*, *G*, *H*, and *I*). Representative blots are shown. *A*, Gag-Syx6 interaction. Gag-FL was coexpressed with Myc-Syx6 or Myc-Syx12 and subjected to immunoprecipitation. *B*, Gag-Syx6 mutant interaction. Gag-FL was coexpressed with Myc-tagged Syx6 domain mutants and subjected to immunoprecipitation. Syx6F, Syx6N, Syx6C, and Syx6*Δ*SNARE indicate the full-length, the N-terminal domain or C-terminal domain of Syx6, and its derivative with deletion of the SNARE domain, respectively. *C*, Syx6 and Gag domains. Syx6F (255 amino acids): Syx6N (116 amino acids); Syx6C (139 amino acids); SNARE (59 amino acids); transmembrane domain (TM) (20 amino acids). Gag (500 amino acids): MA (132 amino acids); CA (231 amino acids); NC (66 amino acids); p6 (52 amino acids). *D*, Gag mutant-Syx6 interaction. Myc-tagged Syx6 was coexpressed with FLAG-tagged Gag fragments (Gag, MACA, MA, Gag*Δ*MA, and GagΔp6) and subjected to immunoprecipitation. *E*, GST pulldown. GST-fused Gag fragments (Gag, MA, CA, p6, and GagΔMA) were purified with glutathione sepharose beads and then incubated with *in vitro*-translated and [^35^S]-labeled Syx6. Representative fluorograph (*top panel*) and Coomassie brilliant blue staining of the GST-Gag fusions (*bottom panel*) are shown. *Dashed line* indicates that one lane (no sample lane) was excised from the original fluorograph. indicates that one lane (no sample lane) was excised from the original fluorograph. *Arrowheads* indicate individual Gag fragments fused with GST. *F*, particle release of GagΔMA in Syx6-depleted cells. 293T cells (in 6-cm dishes) were cotransfected with 1 μg of a pNL43 derivative expressing Gag*Δ*MA and 80 nM Syx6 siRNAs (siSyx6-204 and siSyx6-335) or GFP siRNA (siGFP, control). At 48 h posttransfection, cells and purified HIV-1 particles were analyzed by western blotting using anti-HIV-1 p24CA, anti-Syx6, and anti-actin mAbs. Representative blots are shown. *G*, Gag-SNARE partner interaction. Gag-FL was coexpressed with either Myc-tagged Syx16 (Qa-SNARE), Vti1a (Qb-SNARE), Syx6 (Qc-SNARE), and VAMP4 (R-SNARE) and subjected to immunoprecipitation. A model for spatial distribution of SNAREs and Gag is shown. *H*, formation of SNARE complex composed of Qa-, Qb-, Qc-, and R-SNARE. Myc-tagged Syx16, Vti1a, and Syx6 were together coexpressed with FLAG-tagged VAMP4 and subjected to immunoprecipitation. *I*, Gag-free Syx6 interaction. Gag-FL was coexpressed with Myc-tagged Syx16, Vti1a, Syx6, and VAMP4 and subjected to immunoprecipitation.

Syxs are tail-anchored cytoplasmic proteins that consist of N-terminal hydrophobic regions with coiled-coil structures and C-terminal transmembrane domains. Another coiled-coil structure, termed the SNARE domain, is located near the C-terminal domain and drives SNARE complex assembly (35). The interaction of Gag and Syx6 or Syx12 was examined by coimmunoprecipitation (Fig. 2, *A*–*D*). Gag tagged with the FLAG sequence at the C-terminus (Gag-FL) and Syx6/Syx12 tagged with the Myc sequence at the N-terminus (Myc-Syx) were coexpressed in 293T cells and solubilized with 1% Triton X-100. The samples were subjected to immunoprecipitation with anti-FLAG mAb, followed by western blotting. Gag-FL coprecipitated Myc-Syx6, but not Myc-Syx12 (Fig. 2*A*). To define the domain responsible for the interaction, Syx6 fragments were similarly analyzed. The C-terminal half of Syx6 including the SNARE domain (Syx6C) bound with Gag. Deletion of the SNARE domain (Syx6*Δ*SNARE) abolished Gag binding, suggesting the interaction of Gag with the SNARE domain (Fig. 2, *B* and *C*). The SNARE domain alone was unstable and unavailable for analysis. Using a series of Gag domain constructs tagged with FLAG, we defined the region within Gag necessary for Syx6 binding. The Gag fragments containing the MA domain bound with Syx6, whereas the Gag fragment in which the majority of MA was deleted (Gag*Δ*MA) failed to bind with Syx6 (Fig. 2*D*). To verify the Gag-Syx6 interaction, N-terminal GST-tagged Gag constructs were expressed in *E. coli*, purified, and subjected to GST pulldown assay with *in vitro*-translated [^35^S]Syx6. Syx6 was precipitated with the Gag constructs only when they contained MA (Fig. 2*E*). These results indicate that the Gag MA domain directly interacts with Syx6, independently of Gag membrane binding. Previous studies have shown that a large deletion in MA, when the N-terminal myristoylation signal is intact, does not completely abolish particle production (53, 54). When a pNL43 derivative expressing Gag*Δ*MA was cotransfected with siSyx6 in 293T cells, the levels of particle production were comparable with those in siGFP-cotransfected cells (Fig. 2*F*), indicating that the involvement of Syx6 in HIV-1 particle production is canceled by deletion of MA.

The SNARE proteins are classified into four groups, based on the amino acid sequence homologies (*i.e.*, highly conserved glutamine or arginine) of the SNARE domain. Syxs are generally classified as Qa-SNAREs, but some Syxs are Qc-SNAREs. Soluble N-ethylmaleimide-sensitive factor attachment protein (SNAP) and Vti1 proteins are Qb- and Qc-SNAREs. VAMP proteins are all R-SNAREs (49). We tested if Gag interacted with a binding partner(s) of Syx6-mediated SNARE complex (Syx16 as Qa, Vti1a as Qb, Syx6 as Qc, and VAMP4 as R), although some other combinations were possible (see Discussion). Myc-tagged Syx16, Vti1a, Syx6, and VAMP4 were coexpressed with Gag-FL in 293T cells and were subjected to immunoprecipitation with anti-FLAG mAb. Gag bound with Syx6 but not with Syx16, Vti1a, or VAMP4 (Fig. 2*G*).

One vesicle membrane-associated R-SNARE (v-SNARE) and three target membrane-associated Q-SNARE (t-SNARE) form a highly organized *trans*-SNARE complex, leading to membrane fusion (35, 49, 55). We coexpressed FLAG-tagged R-SNARE (FL-VAMP4) together with Myc-tagged Qa-, Qb-, and Qc-SNAREs (Myc-Syx16+Myc-Vti1a+Myc-Syx6), and immunoprecipitated with anti-FLAG mAb followed by western blotting. Expression levels of individual SNARE molecules varied, but nearly equivalent levels of the SNARE molecules were precipitated under high stringent wash conditions (>1% TritonX-100), suggesting that stable and stoichiometric complexes formed (Fig. 2*H*). We next coexpressed Gag-FL together with all the Qa-, Qb-, Qc-, and R-SNAREs (all tagged with the Myc sequence) and similarly analyzed by immunoprecipitation. Gag predominantly precipitated Syx6, but very little of any other molecules, suggesting that Gag preferentially binds to free Syx6 but is not incorporated into the QabcR-SNARE complex (Fig. 2*I*).

### Gag is localized to and transported by Syx6-positive compartments/vesicles

**Figure 3:**
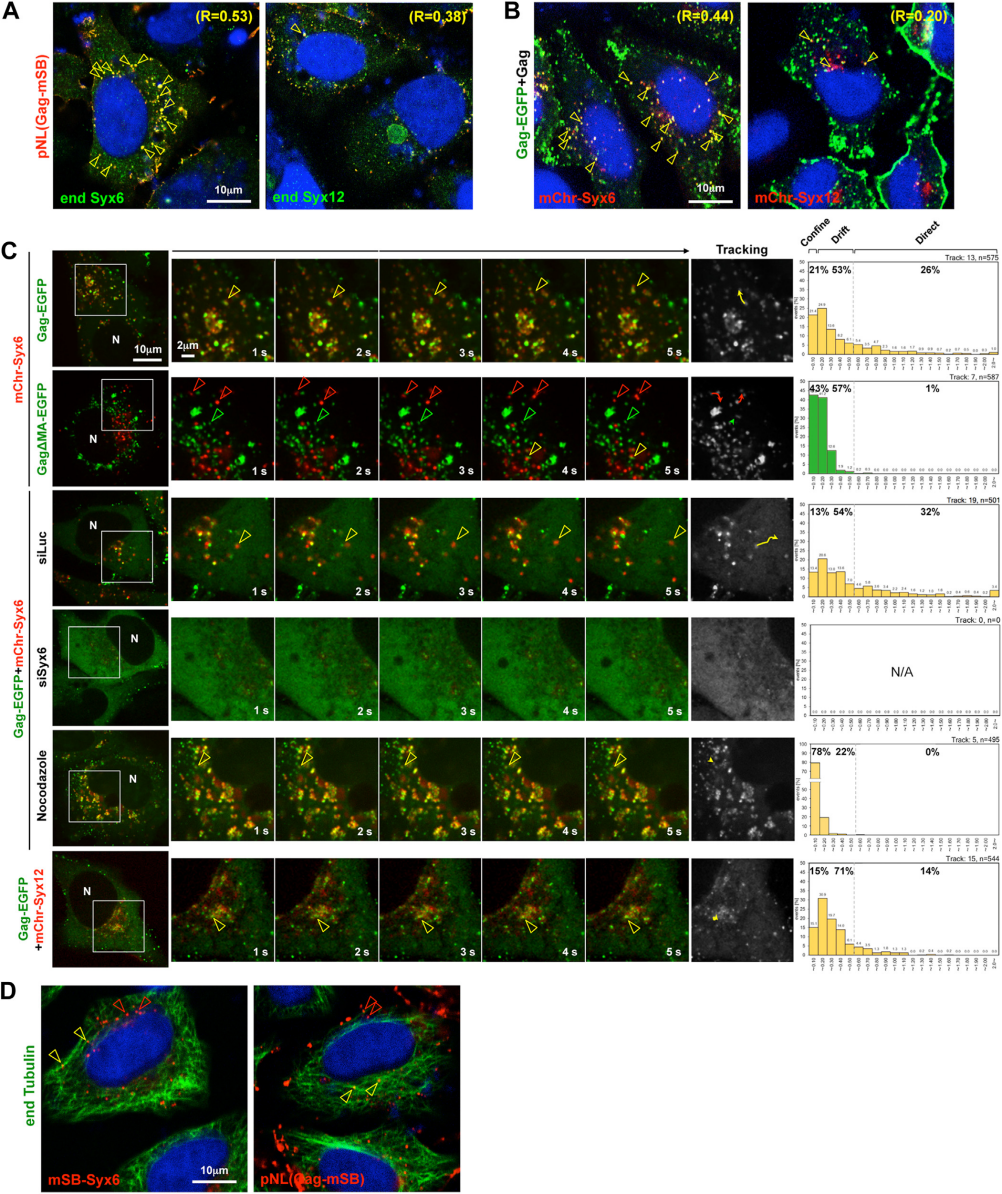
**Gag colocalizes and is cotransported with Syx6.***A*, confocal images of Gag and endogenous Syx6 and Syx12. HeLa cells were transfected with pNL43 expressing Gag-mSB. At 18 to 20 h posttransfection, cells were fixed and costained with anti-Syx6 or anti-Syx12/13 mAbs. *B*, confocal images of Gag and exogenously expressed Syx6. HeLa cells were cotransfected with Gag-EGFP expression plasmid and mChr-Syx6 or mChr-Syx12 expression plasmid. Nuclei were stained with DAPI. Representative confocal images are shown at the same magnification. *Yellow arrowheads* indicate colocalization of antigens. R indicates Pearson’s correlation coefficient of the antigens. *C*, live-cell imaging of Gag and Syx6. HeLa cells were cotransfected with expression plasmids for Gag-EGFP and mChr-Syx6 (first row), for GagΔMA and mChr-Syx6 (second row), for Gag-EGFP and mChr-Syx12 (sixth row), or were pretreated with 80 nM luciferase siRNA (siLuc, control) (third row) or Syx6 siRNA (siSyx6-335) (fourth row) and cotransfected with expression plasmids for Gag-EGFP and mChr-Syx6. HeLa cells were also cotransfected with expression plasmids for mChr-Syx6 and Gag-EGFP and then treated with 10 μg/ml nocodazole (fifth row). At 20 to 24 h posttransfection, dual color images were acquired at 1-s intervals. N, nuclei. Sequential merged images were shown in Movie S1. Time-split 5 images of each movie (cropped area) are shown. *Yellow arrowheads* indicate cotrafficking signals, whereas *green* and *red* arrowheads indicate single-colored signals respectively. Trajectories of trafficking signals are shown in *grey* color images. Velocities of Gag-EGFP signals cotransported with Syx6 signals (first, third, and fifth rows) and cotransported with Syx12 signals (sixth row) are shown as histograms. *D*, confocal images of Gag and Syx6 with microtubules. HeLa cells were transfected with mSB-Syx6 expression plasmid (*left*) or pNL43 expressing Gag-mSB (*right*) and immunostained with anti-α-tubulin mAb. *Yellow arrowheads* indicate mSB-Syx6 or Gag-mSB signals along microtubules, whereas *red arrowheads* indicate mSB-Syx6 or Gag-mSB signals, not associated with microtubules.

To investigate whether Gag was localized to Syx6-and Syx12-positive compartments/vesicles, HeLa cells were transfected with a pNL43 derivative expressing Gag-mStrawberry (mSB) and were immunostained for endogenous Syx6 and Syx12 at early time points (18–20 h posttransfection). Gag-mSB frequently colocalized with Syx6 but much less with Syx12 (the mean Pearson’s R = 0.53 and 0.38, respectively) (Fig. 3*A*). When Gag-EGFP (plus untagged Gag) were coexpressed with mCherry (mChr)-Syx6 or mChr-Syx12, Gag-EGFP partially colocalized with mChr-Syx6 but rarely colocalized with mChr-Syx12 (the mean R = 0.44 and 0.20, respectively) (Fig. 3*B*). Similar results were observed in 293T cells (Fig. S3, *C* and *D*); however, HeLa cells were easier to observe intracellular antigen localization because of a large cytoplasmic space.

Syx6 is localized to the TGN and the EE, but is not compartment-resident, suggesting that Syx6 takes part in various membrane fusion events in post-Golgi trafficking pathways (38). In fact, a previous study revealed the dynamic movements of post-Golgi SNAREs (Syx6 and Syx12)-loaded vesicles/compartments by live-cell imaging (37). We employed live-cell imaging to explore whether Gag was cotransported with Syx6 in the cytoplasm. Confocal dual-color imaging revealed that the majority of Gag-EGFP signals colocalized with mChr-Syx6 signals and moved together (Fig. 3*C*, first row and Movie S1). Tracking of the double-positive signals showed that the velocities of motile events ranged from 0 to >2.0 μm/s. A previous trajectory analysis of adenovirus has identified several different motions: confined (<0.1 μm diameter); drifts (<0.5 μm/s); directed/rapid (>0.5 μm/s) (56). Based on this category, the majority of Gag-EGFP and mChr-Syx6 double-positive signals was in drift (0.1–0.5 μm/s, 53%), and the rapid motile events (>0.5 μm/s), most likely movement along the microtubule (31, 57, 58, 59), were in one-quarter of the population (26%). The Gag-EGFP and mChr-Syx6 double-positive signals, when are additionally coexpressed with untagged Gag, showed a similar velocity profile (Movie S1, 0.1–0.5 μm/s, 57%). When Gag*Δ*MA-EGFP was coexpressed with mChr-Syx6, their signals were almost completely segregated. Individual tracking analysis showed that the motion of Gag*Δ*MA-EGFP signals was confined or drifted (43% and 57%, respectively), and little or no rapid motion was seen (Fig. 3*C*, second row and Movie S1), suggesting that MA is responsible for the Gag-Syx6 colocalization and for the rapid trafficking of Gag. In contrast, the velocities of mChr-Syx6 signals were similar to those of the cotrafficking signals of Gag-EGFP and mChr-Syx6. Next, the motion of Gag-EGFP signals was analyzed in Syx6-knockdown cells. In non-cognate siRNA (siLuc)-treated cells, Gag-EGFP and mChr-Syx6 double-positive signals moved together. The velocities of the double-positive signals showed profiles similar to those in the absence of siRNA (drift, 54%; rapid, 32%) (Fig. 3*C*, third row and Movie S1). In siSyx6-treated cells, no apparent mChr-Syx6 signals were seen, and Gag-EGFP was immotile and diffusely distributed throughout the cytoplasm (Fig. 3*C*, fourth row and Movie S1). For comparison, Gag-EGFP was coexpressed with mChr-Syx12. Consistent with the images of paraformaldehyde-fixed cells (Fig. 3*B*), Gag-EGFP signals rarely colocalized with mChr-Syx12 but some moved in association with mChr-Syx12-positive compartments (Fig. 3*C*, sixth row and Movie S1).

To clarify whether the rapid motion of the Gag-Syx6 complex was microtubule-dependent, Gag-EGFP and mChr-Syx6 were coexpressed and the cells were treated with nocodazole. Live-cell imaging and subsequent tracking analysis revealed that the majority of the Gag-EGFP and mChr-Syx6 double-positive signals was confined, and no rapid movement was seen (78% and 0%, respectively) (Fig. 3*C*, fifth row and Movie S2). However, a fraction of the double-positive signals still moved slowly (drift, 22%), suggesting that Syx6 is responsible not only for rapid, microtubule-dependent trafficking but also for slow, microtubule-independent motion of Gag. When cells expressing Gag-mSB or mSB-Syx6 were immunostained for endogenous tubulin, some, but not all, of Gag-mSB and mSB-Syx6 signals were observed along microtubules (Fig. 3*D*).

### Interaction of Gag with Syx6 *via* the fifth α-helix (H5) of MA is responsible for Gag transport

**Figure 4:**
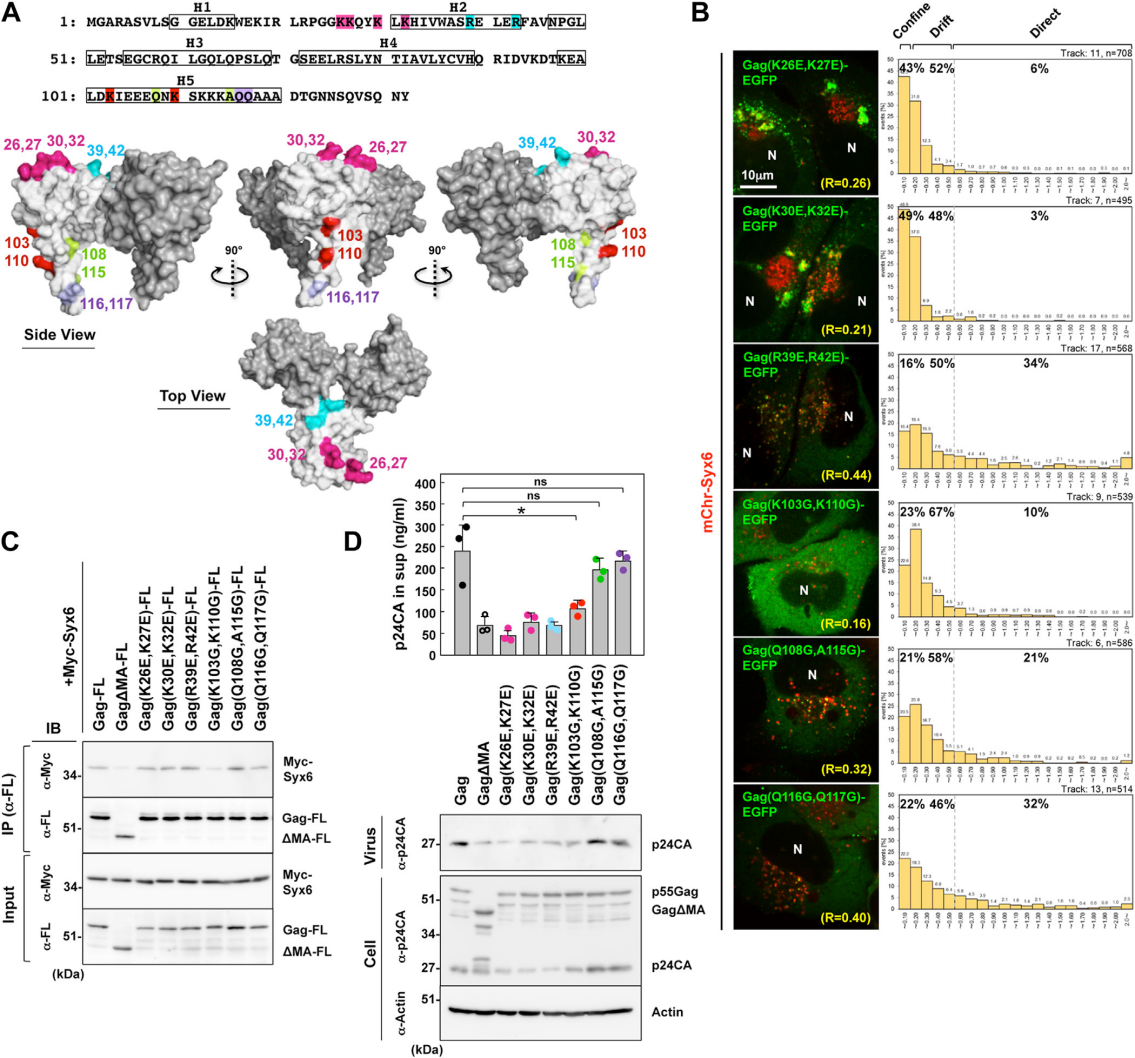
**The H5 region of MA is responsible for the Gag-Syx6 interaction and cotrafficking.***A*, the amino acid sequence of MA and structure of an MA trimer. Based on the coordinates of Gag MA trimer (PDB accession number 1HIW, https://doi.org/10.2210/pdb1HIW/pdb), the protein surface model of an MA trimer (one is shown in *white* and the other two in *grey*) was generated with PyMOL (The PyMOL molecular graphics system, version 2.2.0, Schrödinger, LLC). The amino acid substitutions tested in this study (colored). H1-5, helix1-5. *B*, live-cell imaging of Gag MA mutants with mChr-Syx6 was performed as described in the legend for Figure 3*C*. R indicates Pearson’s correlation coefficient of the antigens. *C*, the Gag MA mutant-Syx6 interaction. Sample solubilization and coimmunoprecipitation were performed as described in the legend for Figure 2. *D*, HIV-1 particle release. HeLa cells (in 6-cm dishes) were transfected with 1 μg of pNL43 derivatives containing Gag MA mutations. The cells and purified HIV-1 particles were analyzed by western blotting using anti-HIV-1 p24CA and anti-actin mAbs. HIV-1 particle yields were quantified by HIV-1 p24CA antigen ELISA. Data are the mean with SD from 3 independent experiments. ∗*p* < 0.05; ns, not significant, Mann-Whiteny U test.

The Gag MA domain is composed of five α-helices termed H1-H5. The H1-H4 tightly associate with each other to form a globular head domain. The H5 extends from the head domain to the Gag CA domain. The MA domain forms a trimer and basic amino acids are clustered at the N-terminal surface of the trimer, regulating membrane binding of Gag (60, 61). Point mutants in the surface of an MA trimer were generated and were expressed in the context of Gag-EGFP (Fig. 4*A*). They were coexpressed with mChr-Syx6 and their trafficking was analyzed by live-cell imaging. Previous studies have shown that the K26E,K27E and K30E,K32E mutants whose residues are located within the HBR, fail to bind to PI(4,5)P_2_ and mislocalize to the LE/MVB (3, 4, 62, 63, 64). These mutants partially colocalized with mChr-Syx6 (the mean Pearson’s R = 0.26 and 0.21, respectively) but were either immotile (*i.e.*, confined) or moved slowly (Fig. 4*B* and Movie S3). A substantial fraction of the mutant Gag-EGFP appeared as large aggregates, consistent with previous results showing particle budding to the LE/MVB (62, 63). In contrast, Gag-EGFP containing R39E,R42E located in the center of the MA trimer, efficiently colocalized with mChr-Syx6 (the mean Pearson’s R = 0.44), and moved together at velocities similar to those of the WT Gag-EGFP and mChr-Syx6 (Fig. 4*B* and Movie S3).

Since the H5 of the MA domain is located at the outer side of an MA trimer, we generated their point mutants and analyzed by live-cell imaging. Double point mutations we introduced matched the 3.6 residues per turn of α-helix, and thus lined up on the surface of the H5. The Q108G,A115G, and Q116G,Q117G Gag-EGFP showed a phenotype similar to the WT Gag-EGFP. They colocalized with mChr-Syx6 (the mean Pearson’s R = 0.32 and 0.40, respectively) and moved together at velocities similar to those of the WT Gag-EGFP (Fig. 4*B* and Movie S3). In contrast, the majority of Gag-EGFP with K103G,K110G mutation exhibited diffuse distribution throughout the cytoplasm, with little colocalization with mChr-Syx6 (the mean Pearson’s R = 0.16) (Fig. 4*B* and Movie S3), suggesting the involvement of basic amino acids on the outer surface of H5 in the MA-Syx6 interaction, although the K103G,K110G mutation alone was insufficient for complete block in the interaction.

The interaction of these MA mutants and Syx6 was examined by coimmunoprecipitation. Gag-FL containing the MA mutations and Myc-Syx6 were coexpressed in 293T cells, solubilized with 1% Triton X-100, and then subjected to immunoprecipitation with anti-FLAG mAb, followed by western blotting (Fig. 4*C*). Only the K103G,K110G mutation reduced the level of coprecipitated Myc-Syx6 to a level similar to Gag*Δ*MA (Fig. 4*C*). The mutations of basic amino acids in the membrane-binding region (K26E,K27E, K30E,K32E, and R39E,R42E) did not apparently impair the Gag-Syx6 interaction. These Gag mutants were expressed in the context of HIV-1 clone pNL43 and p24CA antigens released to the culture medium were quantified (Fig. 4*D*). The K103G,K110G mutation reduced viral particle production (45% compared with the WT). The mutations K26E,K27E, K30E,K32E, and R39E,R42E had slightly more impact on reduction in particle production (<40% compared with the WT), consistent with previous results (62). Thus, the MA-Syx6 interaction was responsible for intracellular Gag trafficking with Syx6, but the Gag membrane binding was not involved in the MA-Syx6 interaction.

As shown in Fig. S5, the SNARE domain forms a long α-helix (16 turns) that is largely hydrophobic, but charged amino acids are clustered on the surface of the helix. Similarly, charged amino acids are clustered on the H5 of MA and its flanking regions (Fig. S5, *A* and *B*). The charged amino acids of H5 and flanking regions were mutated in the context of Gag-FL and their binding abilities to Myc-Syx6 were analyzed by co-immunoprecipitation. The R91G, D93G, E99G, and E105G mutations, when combined with K110G, impaired the Gag-Syx6 interaction (Fig. S5*C*, left). The K103G,E106G mutation reduced the interaction. These amino acids were located from the proximity of the membrane to the outer surface of H5 in an MA trimer (Fig. S5*C*, right), suggesting that electrostatic interactions contribute to the MA-SNARE domain interaction.

### Gag and Syx6 partially colocalize in the EE and recycling endosomes (RE)

**Figure 5:**
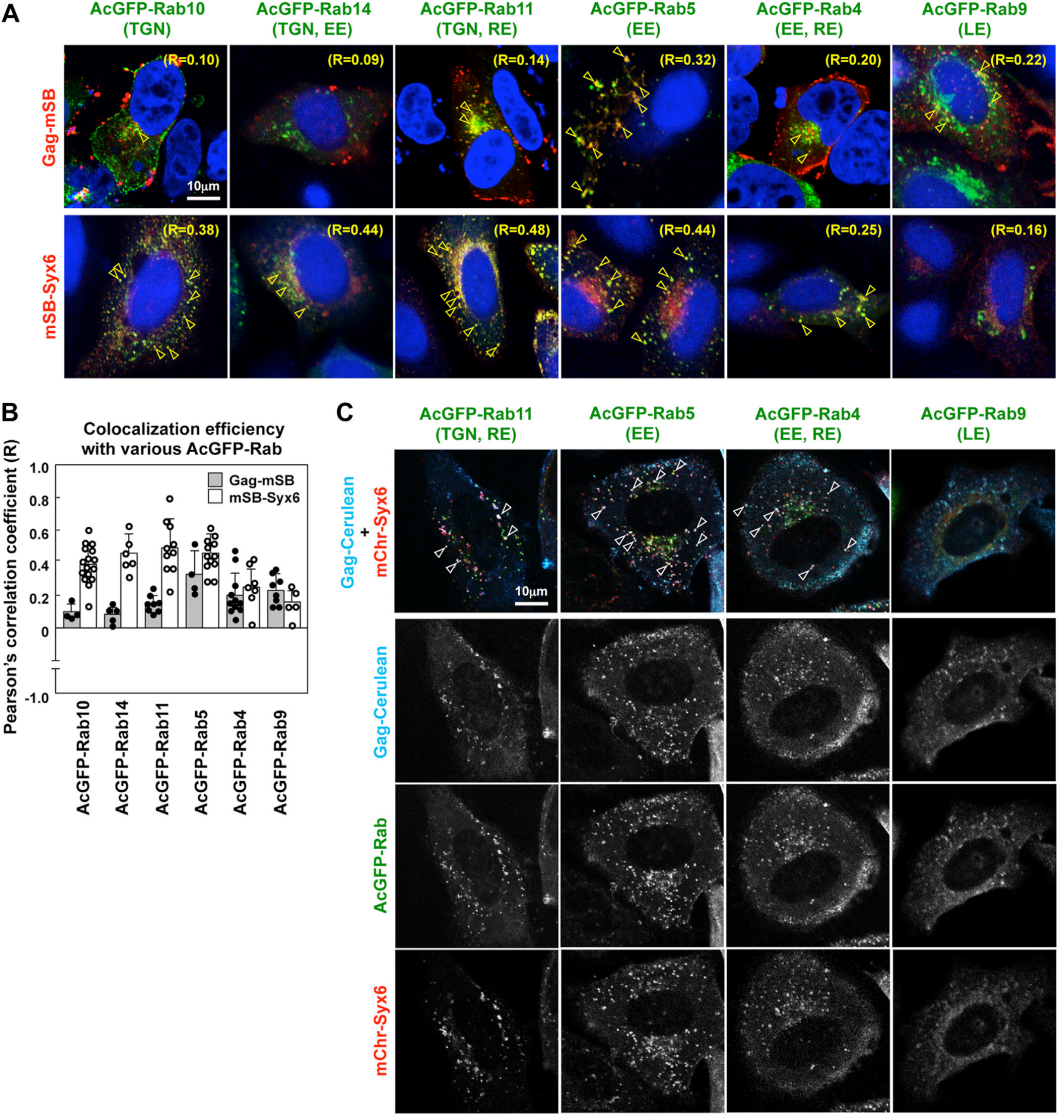
**Gag and Syx6 colocalize in the EE and the RE.***A*, intracellular localization of Gag and Syx6. HeLa cells were cotransfected with Gag-mSB (*upper*) or mSB-Syx6 (*lower*) expression plasmid and various AcGFP-Rab protein expression plasmids (indicated). At 24 h posttransfection, cells were fixed and subjected to confocal microscopy. Nuclei were stained with DAPI. Representative confocal images are shown at the same magnification. *Arrowheads* indicate colocalization of antigens. R indicates Pearson’s correlation coefficient of the antigens. *B*, colocalization efficiency of Gag or Syx6 with various AcGFP-Rab proteins. Pearson’s correlation coefficients of the two antigens (the mean with SD) are shown. *C*, colocalization of Gag and Syx6 in RabX-positive compartments. Gag-Cerulean, AcGFP-RabX, and mChr-Syx6 were triply coexpressed in HeLa cells. At 24 h postinfection, cells were fixed and subjected to confocal microscopy. *Arrowheads indicate* triple colocalization of Gag-Cerulean, AcGFP-RabX, and mChr-Syx6. Each channel image is shown in *gray*.

Syx6 is found to be involved in multiple trafficking pathways through the association with various SNARE proteins (38, 65). To explore the Syx6-mediated Gag transport pathways, we used AcGFP-Rab proteins as markers for subcellular localization, since they are distributed in their specific target compartments: Rab10 and Rab14 predominantly in the Golgi and TGN; Rab11 in the TGN and RE; Rab5 in the EE; Rab4 in the EE and RE; Rab9 in the LE (66, 67). mSB-Syx6 was coexpressed with various AcGFP-Rab proteins in HeLa cells and was observed by confocal microscopy (Fig. 5*A*, lower). mSB-Syx6 frequently colocalized with AcGFP-Rab10, Rab14, and Rab11 (the mean Pearson’s R = 0.38, 0.44, and 0.48, respectively). Colocalization was also observed with AcGFP-Rab5 and Rab4 (the mean R = 0.44 and 0.25, respectively). In contrast, little colocalization was seen for Rab9 (the mean R = 0.16). These data indicate that Syx6 is broadly distributed in post-Golgi biosynthetic pathways including the EE and RE, consistent with previous studies (38, 65). Next, Gag-mSB was coexpressed with the AcGFP-Rab proteins and observed by confocal microscopy (Fig. 5*A*, upper). Gag-mSB rarely colocalized with AcGFP-Rab10, Rab14, and Rab11 (the mean R = 0.10, 0.09, and 0.14, respectively) but partially colocalized with AcGFP-Rab5, Rab4, and Rab9 (the mean R = 0.32, 0.20, and 0.22, respectively), indicating that Gag is preferentially distributed to the endocytic pathways. These data potentially suggest that Gag colocalizes with Syx6 in the EE and RE pathways (Fig. 5*B*). To test this hypothesis, Gag-Cerulean, AcGFP-Rab, and mChr-Syx6 were triply coexpressed and their colocalization was observed by confocal microscopy (Fig. 5*C*). Gag-Cerulean and mChr-Syx6 partially colocalize in AcGFP-Rab11-, -Rab5-, and -Rab4-positive compartments. In contrast, colocalization was barely seen in AcGFP-Rab9-positive compartments.

### Syx6 is responsible for HIV-1 replication and particle production in immune cells

**Figure 6:**
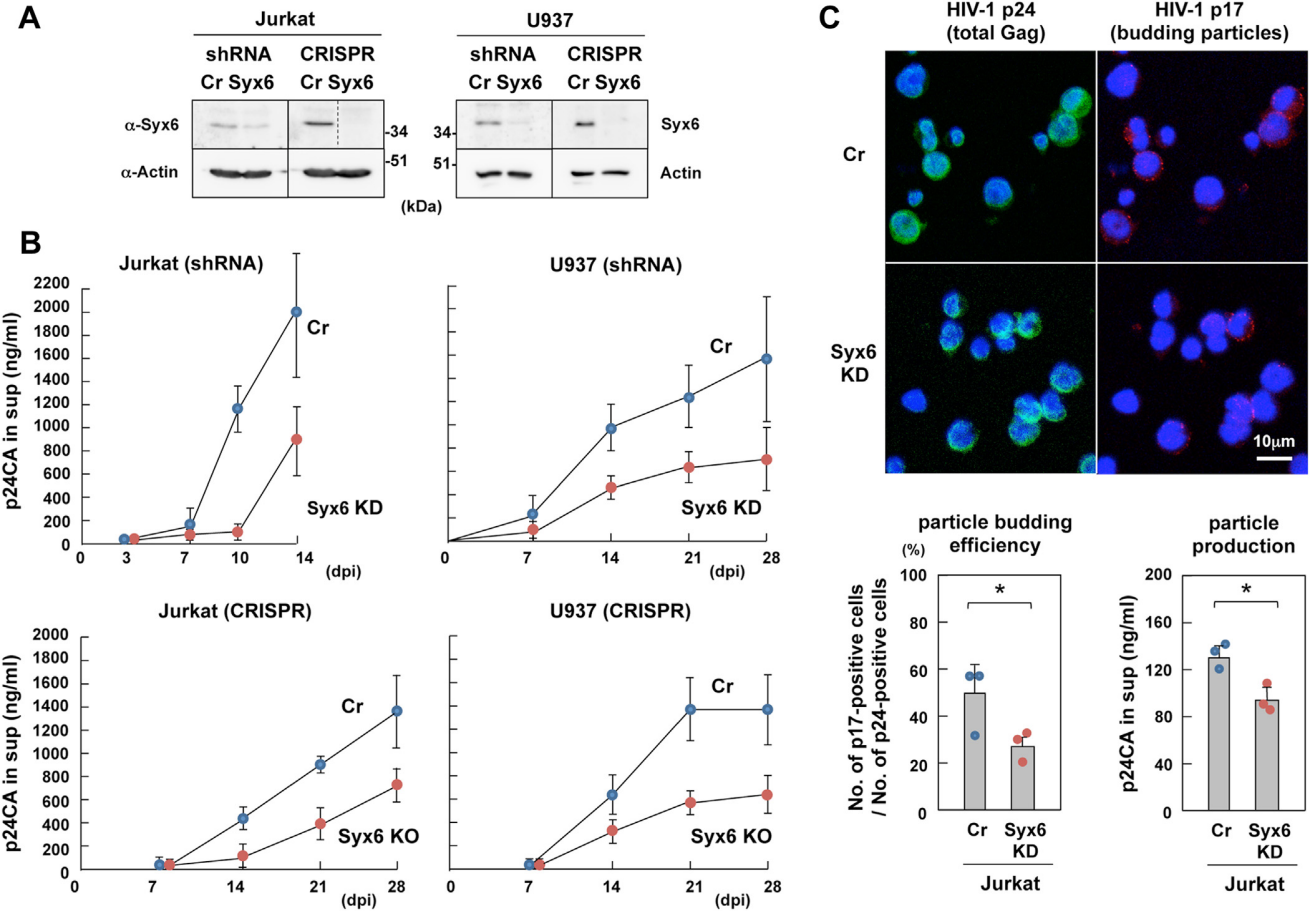
**Knockdown and knockout of Syx6 impair HIV-1 replication in human T cell and monocytic cell lines.***A*, Jurkat and U937 cells transduced with lentiviruses expressing 3 distinct Syx6 shRNAs or scramble control shRNAs were subjected to western blotting with anti-Syx6 and anti-actin mAbs. Cells transduced with lentivirus expressing Syx6 gRNA (targeting nucleotide 553–572 of Syx6 ORF) and Cas9 were cloned and then analyzed. Cells transduced lentivirus expressing Cas9 alone were used as control. A *dashed line* indicates that one lane (the Syx6 gRNA-transduced cell mixtures) was excised from the original blot. *B*, HIV-1 replication. The Syx6-knockdown/knockout Jurkat (*left*) and U937 cells (*right*) were infected with HIV-1 at an MOI of 0.1. HIV-1 particle yields were temporally quantified by HIV-1 p24CA antigen ELISA. Data are the mean with SD from 3 independent experiments. *C*, HIV-1 particle release from Syx6-knockdown Jurkat cells. Syx6-knockdown Jurkat cells and scramble control cells were infected with HIV-1 at MOI of 5. At 48 h postinfection, the cells were immunostrained with anti-HIV-1 p17MA mAb specific for the mature p17MA C-terminal epitope, followed by Alexa Fluor 568-conjugated anti-mouse IgG and costained with FITC-labeled anti-HIV-1 p24CA mAb. More than 350 cells were subjected to analysis in each group. The efficiency of particle budding was assessed as the ratio of the number of p17MA-positive cells to that of p24CA-positive cells (*lower left*). Levels of p24CA antigens in the culture media were quantified by ELISA (*lower right*). The mean with SD from 3 independent experiments. ∗*p* < 0.05, ns, not significant, Mann-Whiteny U test.

To explore whether Syx6 was responsible for HIV-1 production in immune cells, Jurkat and U937 cells were transduced with lentiviruses expressing shRNAs targeting Syx6 (for knockdown) and expressing Cas9 and gRNAs targeting Syx6 (for knockout) and stable cell lines were established. The knockdown and knockout of Syx6 were confirmed by western blotting (Fig. 6*A*). These cells were infected with HIV-1 (the NL strain) at low multiplicity of infection (MOI) and HIV-1 replication was monitored by p24CA ELISA (Fig. 6*B*). In both Jurkat and U937 cell lineages, Syx6 knockdown (Fig. 6*B*, upper) and knockout (Fig. 6*B*, lower) delayed HIV-1 replication and reduced virus yields compared with their control cells, although HIV-1 replication was not completely abrogated. In our study, the knockout cell systems showed slow/delayed virus replication compared with the knockdown cell systems. This is possibly due to genetic compensation mechanisms upon nonsense-mediated RNA decay as suggested in zebrafish studies (68) or cell viability altered by constitutive expression of the Cas9 enzyme in the knockout cells.

Syx6 knockdown Jurkat cells were infected with HIV-1 at high MOI and immunostained for Gag antigens (p24CA for total Gags and p17MA for budding particles) at 2 days postinfection. We used the anti-p17MA mAb that recognizes only the mature p17MA domain produced upon HIV-1 particle budding to detect particle-producing cells (62, 63). The p24CA antigens were very frequently observed in both Syx6 knockdown and scramble shRNA-transduced control cells (>80% in both cells). The p17MA antigens were frequently observed in the control cells but were less evident in Syx6 knockdown cells (Fig. 6*C*, panels). Particle budding efficiency was assessed by the percentages of p17MA-positive cells in the p24CA-positive cells. For a more direct measurement of particle production, the production of p24CA antigens was measured by p24CA ELISA. Syx6 knockdown reduced the p24CA level to 65% compared with the control (Fig. 6*C*, lower). Together, these results indicate that Syx6 is partly responsible for HIV-1 particle production in immune cells.

### Cotransport of TNFα and Gag *via* Syx6-positive compartments/vesicles

**Figure 7:**
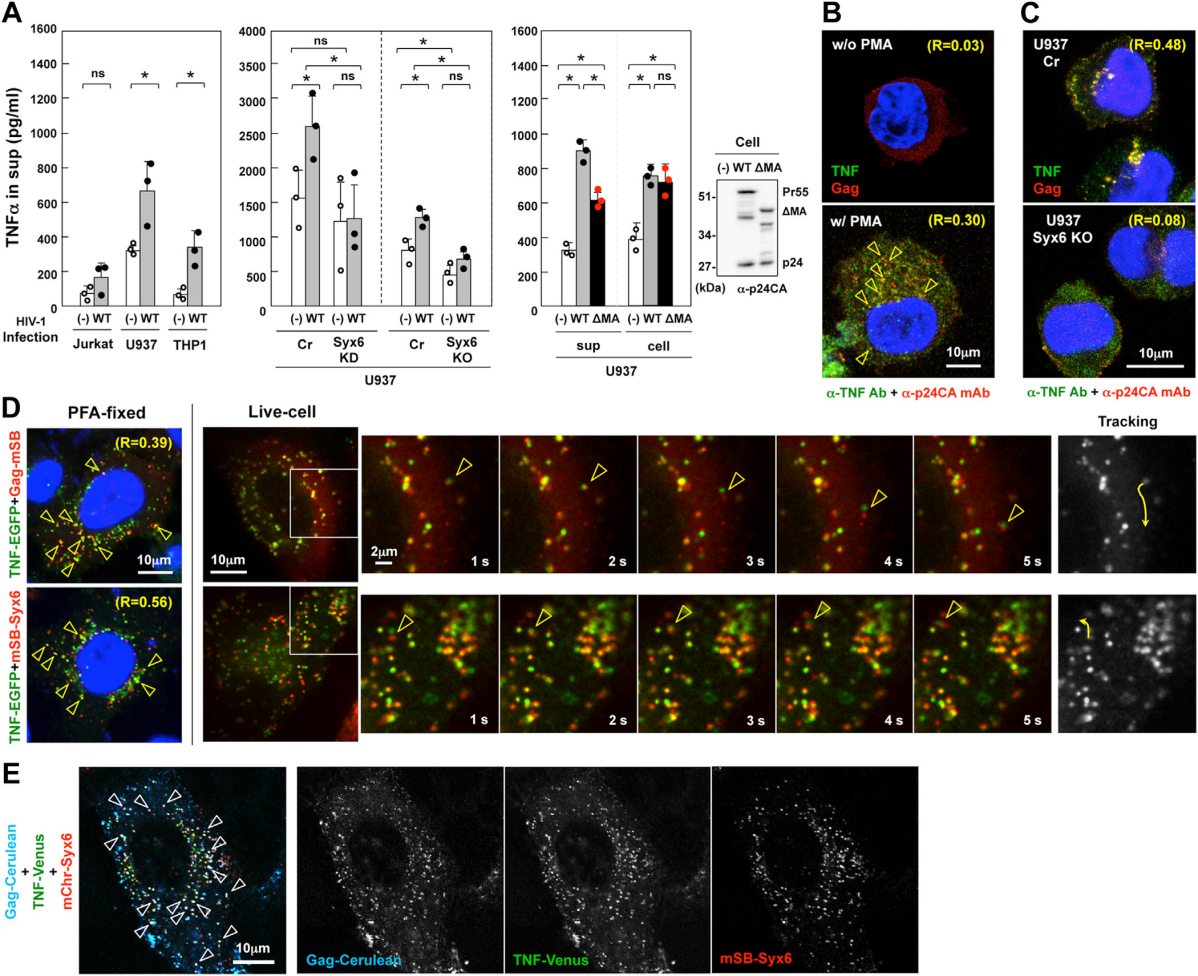
**Cotrafficking of Gag and TNFα *via* Syx6-positive compartments/vesicles.***A*, TNFα secretion. (*Left*) Jurkat, U937, and THP-1 cells (2–3 × 10^5^ cells/ml) were treated with 100 ng/ml PMA and uninfected (−) or infected with HIV-1 (WT) at MOI of 2. (*Middle*) Syx6-knockdown/knockout U937 cells were similarly treated with PMA and uninfected or infected with HIV-1. (*Right*) U937 cells were treated with PMA and infected with HIV-1 (WT) or MA-deleted HIV-1 (*Δ*MA). TNFα secretion in the culture medium (at 48 h postinfection) was monitored by ELISA. The cells were lysed (in 3 × 10^5^ cells/ml) and subjected to ELISA for TNFα and western blotting for HIV-1 p24CA. The mean with SD from 3 independent experiments. ∗*p* < 0.05; ns, not significant, Mann–Whiteny *U* test. *B* and *C*, colocalization of Gag and TNFα. *B*, THP-1 cells were untreated or treated with PMA and infected with HIV-1. *Arrowheads* indicate vesicles/compartments with colocalization of Gag and TNFα. *C*, Syx6-knockout U937 cells were treated with PMA and infected with HIV-1. U937 cells transduced with Cas9 alone were used as control. At 48 h postinfection, cells were immunostained with anti-HIV-1 p24CA mAb and anti-TNFα Ab. *D*, colocalization and cotrafficking of Gag and Syx6 with TNFα. TNF-EGFP was coexpressed with Gag-mSB or mSB-Syx6 in HeLa cells. At 20 to 24 h postinfection, the cells were subjected to live-cell imaging as described in the legend for Figure 3*C*. The cells were also fixed with paraformaldehyde and chilled methanol and observed by a confocal microscope (most *left*). *Arrowheads* indicate cotrafficking signals (in live cells) or colocalized antigens (in fixed cells). R indicates Pearson’s correlation coefficient of the antigens. *E*, colocalization of Gag, TNFα, and Syx6. Gag-Cerulean, TNF-Venus, and mChr-Syx6 were triply coexpressed in HeLa cells. At 24 h postinfection, cells were fixed and subjected to confocal microscopy. *Arrowheads* indicate colocalization of Gag-Cerulean, TNF-Venus, and mChr-Syx6. Each channel image is shown in *gray*.

TNFα is a proinflammatory cytokine that plays a critical role in HIV-1 pathogenesis (69). In clinical settings, the level of TNFα positively correlates with plasma viral load and disease progression (70). Some studies have suggested that Syx6 mediates secretory transport of TNFα in immune cells (71, 72, 73). Thus, it is possible that Gag transport by Syx6 may affect TNFα secretion. To test this possibility, cells were differentiated by treatment with phorbol myristate acetate (PMA) (74, 75) and infected with HIV-1. HIV-1 infection increased TNFα secretion in Jurkat, U937, and THP-1 cells. This was apparent in monocytic U937 and THP-1 but not lymphatic Jurkat cells (Fig. 7*A*, left). Syx6 knockdown and knockout U937 cells were infected with HIV-1 and the levels of TNFα secretion were compared with their control cells. In the control cells, TNFα secretion was enhanced upon HIV-1 infection. In contrast, the enhancement was not observed in Syx6 knockdown or knockout cells (Fig. 7*A*, middle). These results suggest that the upregulation of TNFα secretion upon HIV-1 infection was abrogated by Syx6 depletion. To further explore whether the Gag-Syx6 interaction was involved in the increase of TNFα secretion, U937 cells were infected with HIV-1 expressing the WT Gag or Gag*Δ*MA, and the levels of TNFα in the cells and in culture media were quantified (Fig. 7*A*, right). HIV-1 infection, whether its Gag was WT or *Δ*MA, increased the expression of TNFα to similar levels, suggesting that HIV-1 elements other than Gag was responsible for the upregulation of TNFα expression. However, the TNFα level secreted to the medium was higher in the cells infected with HIV-1 expressing the WT Gag than that expressing Gag*Δ*MA, suggesting that the WT Gag facilitated TNFα trafficking more efficiently than Gag*Δ*MA (Fig. 7*A*, right).

TNFα is transported from the TGN to the PM *via* partly Syx6-mediated pathways (71). To explore if the trafficking pathways of TNFα and Gag overlapped, THP-1 cells were infected with HIV-1 and immunostained with anti-TNFα antibody (Ab) and anti-HIV-1 p24CA mAb. Confocal microscopy revealed partial colocalization of TNFα and Gag (Fig. 7*B*). In U937 cells, they colocalized predominantly at the perinuclear region and partly at the PM. However, when Syx6 was depleted, TNFα was broadly distributed in the cytoplasm (Fig. 7*C*).

Colocalization of TNFα with Gag and Syx6 was explored in HeLa cells where TNFα-EGFP was coexpressed with Gag-mSB or mSB-Syx6. Confocal microscopy revealed that TNFα-EGFP frequently colocalized with Gag-mSB and mSB-Syx6 (the mean Pearson’s R = 0.39 and 0.56, respectively) (Fig. 7*D*, most left). Dual-color live-cell imaging revealed that a faction of TNFα-EGFP colocalized with Gag-mSB and with mSB-Syx6, and moved together (Fig. 7*D*, right images and Movie S4). Finally, Gag-Cerulean, TNFα-Venus, and mChr-Syx6 were triply coexpressed and their colocalization was observed. Confocal images indicated that the three antigens often colocalized in the cytoplasm (Fig. 7*E*). Together, the data suggest a possibility that Gag and TNFα are cotransported *via* Syx6-positive compartments/vesicles.

### TNFα directly binds to Syx6 but not to Gag

**Figure 8:**
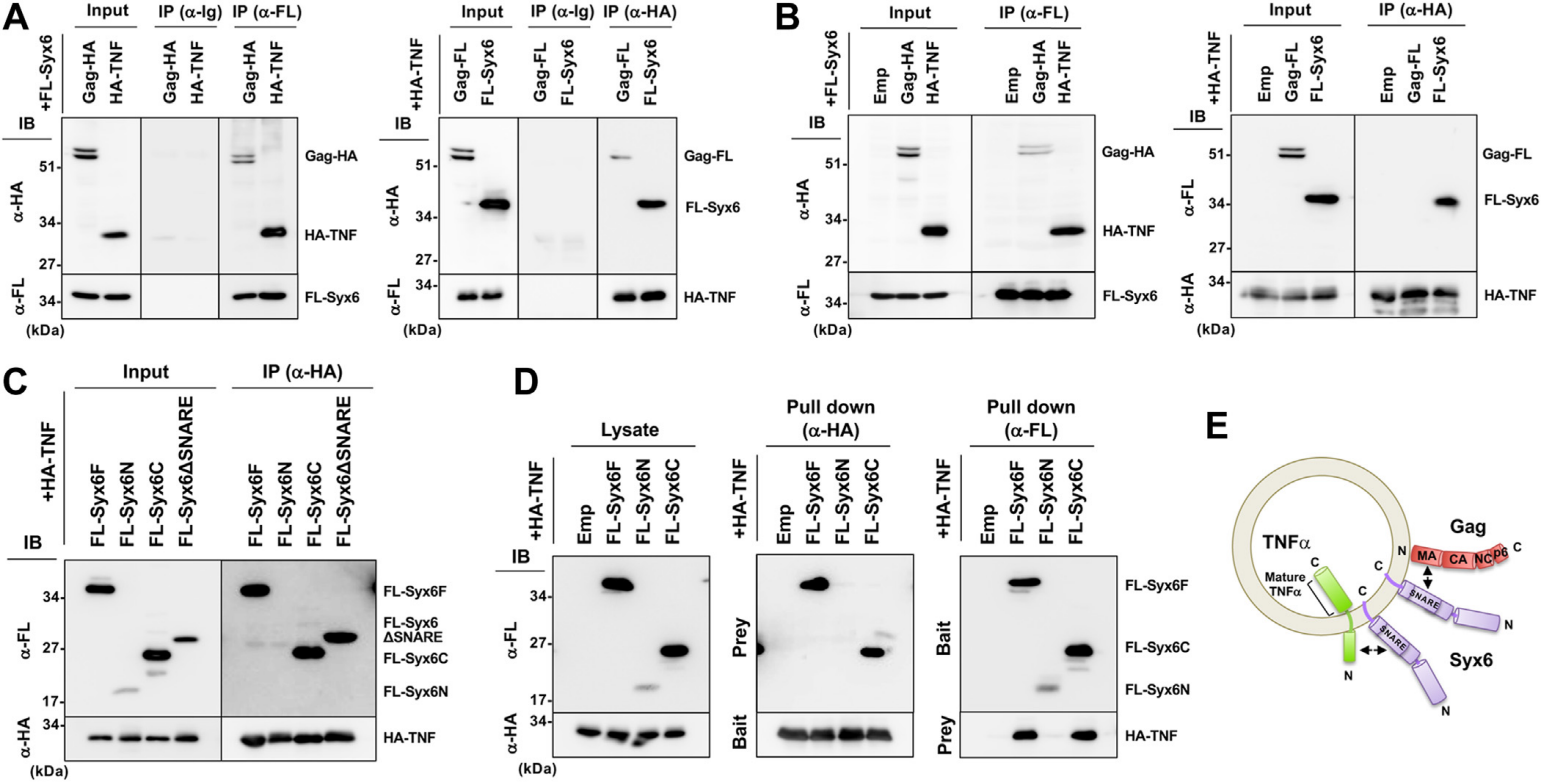
**TNFα directly binds with Syx6, whereas Gag associates with TNFα *via* Syx6-positive membrane.** Constructs were expressed in 293T cells and subjected to immunoprecipitation with anti-FLAG and anti-HA mAbs, followed by western blotting with anti-FLAG and anti-HA mAbs (*A*–*C*). Representative blots were shown. *A*, association of Gag, Syx6, and TNFα through the membrane. Following membrane flotation centrifugation, membrane-bound fractions were subjected to immunoprecipitation. Normal mouse IgG was used as a negative control. *B*, interaction among Gag, Syx6, and TNF. Cells were lysed in lysis buffer containing 1% Triton-X 100 and subjected to immunoprecipitation. *C*, TNFα-Syx6 mutant interaction. HA-TNF was coexpressed with FLAG-tagged Syx6 domain fragments (Syx6F, Syx6N, Syx6C, and Syx6*Δ*SNARE) and analyzed by immunoprecipitation. Syx6F, Syx6N, Syx6C, and Syx6*Δ*SNARE indicate the full-length, the N-terminal domain or C-terminal domain of Syx6, and its derivative with deletion of the SNARE domain, respectively. *D*, FLAG and HA tag pulldown. HA-TNF was bound to sepharose beads and then incubated with cell lysates expressing FLAG-tagged Syx6 fragments (*middle*). FLAG-tagged Syx6 fragments were bound to agarose or sepharose beads and then incubated with cell lysates expressing HA-TNF (*right*). Precipitates were analyzed by western blotting with anti-FLAG and anti-HA mAbs. *E*, a model for the topology and interaction of Gag, Syx6, and TNFα.

We investigated the association of TNFα with Syx6 and Gag. The FLAG sequence-tagged Syx6 (FL-Syx6) was coexpressed with the HA sequence-tagged Gag (Gag-HA) or TNFα (HA-TNFα) in 293T cells. After membrane flotation centrifugation, the membrane fraction was subjected to immunoisolation with anti-FLAG mAb followed by western blotting. Gag-HA and HA-TNFα were coisolated with FL-Syx6 (Fig. 8*A*, left). When HA-TNFα was coexpressed with Gag-FL and FL-Syx6 and the membrane fraction was immunoisolated with anti-HA mAb, Gag-FL and FL-Syx6 were coisolated with HA-TNFα (Fig. 8*A*, right). Then, similar assays were performed using membrane-solubilized lysates. Gag-HA and HA-TNFα were coprecipitated with FL-Syx6 (Fig. 8*B*, left). In contrast, FL-Syx6, but not Gag-FL, was coprecipitated with HA-TNFα (Fig. 8*B*, right). These results indicate that TNFα interacts with Syx6 but associates with Gag only through the membrane.

To define the Syx6 domain responsible for the TNFα-Syx6 interaction, FL-Syx6 fragments were similarly subjected to immunoprecipitation. HA-TNFα coprecipitated FL-Syx6C and FL-Syx6*Δ*SNARE, suggesting that, unlike Gag, TNFα interacts with the C-terminal half fragment other than the SNARE domain (Fig. 8*C*). To further verify the TNFα-Syx6 interaction, HA-TNFα (bait) was immunoprecipitated and then incubated with FL-Syx6 fragments (prey) (Fig. 8*D*, middle). Reversely, FL-Syx6 fragments (bait) were purified with anti-FLAG mAb-immobilized beads and then incubated with HA-TNFα (prey) (Fig. 8*D*, right). Results indicated that TNFα bound with Syx6F and Syx6C (Fig. 8*D*), suggesting that TNFα directly binds with the Syx6 C-terminal half. Since TNFα is a type II transmembrane protein, the N-terminal cytoplasmic domain of TNFα most likely interacts with the C-terminal region of Syx6 (Fig. 8*E*).

## Discussion

### Gag cargo trafficking *via* Syx6-mediated pathways

Our results indicate that Gag colocalizes and cotraffics with Syx6 (Fig. 3). When the Syx6-mediated Gag transport pathways were investigated using AcGFP-Rab constructs, Syx6 was broadly distributed in both biosynthetic and endocytic pathways, whereas Gag was preferentially in the endocytic pathways (Fig. 4). Triple color imaging shows that Gag colocalizes with a fraction of Syx6 that is localized to the EE and RE, marked by the presence of Rab5 and Rab4, respectively, but less with a Syx6 fraction present in Rab11-positive RE (Fig. 5). The EE serves as a sorting platform from which cargos are delivered to a variety of endocytic pathways: the LE-mediated lysosomal pathway for degradation; direct and rapid RE pathways back to the PM, which is defined as Rab4-positive compartments; and slow RE pathways, defined as Rab11-positive compartments (76), which are localized to the perinuclear area and often connected to the TGN (77). Our data indicate that a substantial fraction of Gag colocalizes with the Syx6 fractions present in the EE and in Rab4-positive RE (Fig. 5). These findings suggest that a fraction of Gag is transported *via* the Syx6-positive vesicles/compartments in the EE and RE pathways. These pathways are unlikely to be dead-end pathways of Gag even if the observed Gag antigens represent endocytosed Gag because Syx6 positively regulated HIV-1 production in our study. One possibility is that Gag may be rescued from the endocytic degradation pathways *via* Syx6-positive vesicles/membrane domains. We also observed that a fraction of Gag was distributed to the LE, where Syx6 was poorly distributed. It is possible that once endocytosed Gag reaches the LE, the Gag is poorly or no more rescued by Syx6, resulting in a real dead-end product. These models reconcile previous contradictory observations, one of which shows no contribution of the Gag localized to the LE in particle release (17), and the other shows an increase in particle production when cells were treated with endosomal inhibitors (21). It should be noted, however, that Syx6-mediated trafficking is not the sole pathway for Gag because Syx6 knockdown/knockout does not completely abolish HIV-1 production (Figs. 1 and 5).

### Potential mechanisms for Gag transport mediated by Syx6 in post-Golgi and endocytic pathways

Post-Golgi transport is largely mediated by clathrin-coated vesicles, composed of clathrin and adaptor proteins (AP1-4 and GGA). Arf GTPase recruits the adaptors from the cytosol to the membrane, leading to the budding of clathrin-coated vesicles (33). Rab is also involved in this budding step and subsequently regulates vesicle transport along microtubules. The vesicles are tethered to and fused with the target membrane by *trans*-SNARE complex formation (78). Previous studies have suggested that those molecules are involved in Gag localization and/or particle production (27, 28, 29, 79, 80, 81). Our study identified Syx6, which regulates vesicle translocation in post-Golgi networks including endocytic pathways, as a host factor for Gag trafficking. We emphasize that Gag colocalizes and moves with Syx6 together and that in absence of Syx6, Gag diffusely distributes throughout the cytoplasm (Fig. 3). These observations suggest that Syx6 plays a role in sorting or recruiting and subsequently transporting Gag as a carrier. Interestingly, our additional experiments have found that Syx6 binds to AP1μ and AP3μ in yeast two-hybrid assays (data not shown); however, unlike Syx6, AP1μ and AP3μ were almost static, accumulated, and not cotransported with Gag (Movie S5). These observations suggest that AP1- and AP3-positive compartments are not Gag transport carriers although Gag may transiently interact with these compartments. The APμ subunits have been shown to bind to tyrosine-based YXXØ motifs (Ø, a bulky hydrophobic residue), which act as internalization and intracellular sorting signals (34, 82). Syx6 contains a YXXL motif at amino acids 140 to 143 (38), suggesting a possibility that Gag is recruited to the AP1/AP3-positive compartments not only through direct Gag-AP interactions but also through their interaction with Syx6.

Cumulative evidence suggests that Syx6 can be associated with a variety of SNARE proteins (*e.g.*, Syx4, 12, 16, SNAP23, 25, and VAMP2, 4, 7, 8) (38). The flexibility of Syx6 in SNARE pairing may allow a consecutive fusion process through different SNARE pairing, thereby moving to the next compartments. In accordance with this view, the binding affinity of Syx6 with Gag was much weaker than that with its binding SNARE partners in our coimmunoprecipitation analysis (Fig. 2), suggesting that the Gag-Syx6 interaction may not inhibit the formation of the SNARE complex or subsequent membrane fusion. Thus, Syx6 may act as a molecule to deliver Gag to various compartments during Gag transport. Our coimmunoprecipitations also indicated that Gag preferentially bound with free Syx6 and was not incorporated into the t-SNARE (Qabc-SNARE) complex or *trans*/*cis*-SNARE (QabcR-SNARE) complex (Fig. 2). We currently do not know the precise mechanisms by which Gag is spatiotemporally recruited and bound with free Syx6 generated by disassembly of the *cis*-SNARE complex. However, we find from their membrane topology that the N-terminal MA domain of Gag can interact with the SNARE domain of Syx6, both of which are in proximity to the membrane (Figs. 2*G* and 8*E*). Interestingly, NSF and α-SNAP are the most essential regulators for the disassembly of *cis*-SNARE complex (35) and dominant-negative inhibition of NSF causes diffuse distribution of Gag in the cytoplasm (36), a phenotype which is similar to that seen for Syx6 knockdown (Figs. 1*C* and 3*C*). It is possible, although not proven, that blockage of *cis*-SNARE disassembly (releasing free Syx6) is a prerequisite for Gag’s capturing by Syx6.

PI(4,5)P_2_, which is specifically present at the PM, acts as a critical determinant for Gag targeting to the PM (41). This finding fits the current Gag assembly model, in which monomeric Gags arrive and accumulate at the PM (17, 20). However, it remains unclear how Gag finds and approaches PI(4,5)P_2_-enriched PM. Different types of PIPs are produced in their given compartments [*e.g.*, PI(4,5)P_2_ at the PM, PI(3)P at the EE, and PI(4)P at the TGN and the RE]; however, they are virtually interconvertible by specific kinases and phosphatases (83, 84). Several studies suggest that PIPs recruit downstream effectors, including AP and SNARE molecules to facilitate membrane fusion (85, 86, 87). Syx6 is also known to bind to cholesterol, a major constituent of lipid rafts (88).

### Microtubule-dependent and independent Gag trafficking

We observed that a faction of the Gag-EGFP and mChr-Syx6 double-positive signals moved rapidly in inward and outward directions, likely corresponding to microtubule-associated vesicle trafficking (Fig. 3*C* and Movie S2). Since endosomes and lysosomes are known to move along the microtubule networks (89, 90), it is alternatively possible that they are Syx6-positive endosomes containing Gag, although their motion was faster and more active than those observed in the Gag-targeted LE/MVB (Figs. 3 and 4, Movie S3). Disruption of microtubules by nocodazole treatment caused the complete disappearance of the rapid movement; however, some of the double-positive signals still moved although they were slow (Fig. 3*C*). The mechanisms of the microtubule-independent Gag trafficking observed here are currently unknown. However, several lines of evidence suggest that microtubule-independent membrane trafficking occurs through membrane contact of different organelles (*e.g.*, TGN-RE and ER-diverse organelle interactions) (77, 91) and functional maturation of organelles (92) in microtubule-disrupted cells. The motion of the microtubule-independent membrane trafficking is considerably slow (membrane contact in minutes; to destinations in hours). This mode of membrane trafficking is potentially important and may provide clues to the underlying mechanisms of Gag trafficking because some studies have shown that Gag is still targeted to the PM and produces virus particles in microtubule-disrupted cells (17, 18). Interestingly, TNFα is also transported and secreted even in microtubule-disrupted cells (92) and our study finds that TNFα is cotransported with Gag (Fig. 7).

### Interaction of Gag MA mutants with Syx6 and their trafficking

We used multiple Gag MA mutants to characterize Syx6-mediated Gag trafficking (Figs. 4 and S5). The Gag*Δ*MA mutant contains the N-terminal myristoylation signal but lacks the HBR, a determinant of PI(4,5)P_2_ binding. This mutant also lacks the H5, a binding region with Syx6 and thereby segregated from the Syx6 and partially accumulated in the cytoplasm. Gag*Δ*MA reduced but did not abolish particle production, consistent with previous studies with a large deletion in MA (53, 54). More progressive deletion in MA has been shown to alleviate the reduction, showing efficient particle production, possibly due to a constitutive exposure of the N-terminal myristoyl moiety (93). The HBR mutants (K26E,K27E and K30E,K32E) are also deficient for PI(4,5)P_2_ binding ability (3, 4). Unlike Gag*Δ*MA, these mutants bound to Syx6 as efficiently as the WT Gag *in vitro* and partially colocalized with mChr-Syx6, but similarly displayed immotile accumulation of Gag. Previous studies collectively suggest that without a PI(4,5)P_2_ binding determinant, Gag mistargets to intracellular compartments due to non-selective membrane binding of a myristoyl moiety (2, 62, 63). Our data suggest that membrane binding and Syx6 binding are likely to be independent events for Gag but that the Gag binding to Syx6 is insufficient to redirect these HBR mutants to the PM. In the three Gag mutants, rapid movement was rarely seen (Figs. 3 and 4), but the relationship between their membrane binding and microtubule-mediated trafficking is unknown.

We observed that the charged amino acids, located at the outer surface of the MA H5, including its franking region in a MA trimer, were responsible for the Gag-Syx6 interaction but that the double-point mutations were insufficient for the complete block in the interaction (Figs. 4 and S5). These findings suggest that the interaction between Gag and Syx6 may involve multiple amino acids on the outer surface of H5 or a broader region in MA. The SNARE complex is formed through the interaction of 4 SNARE domains, slightly twisted 4-helix bundle structures. The 4-helix bundle is tightly connected by mostly hydrophobic but partly electrostatic interactions (49). It is very likely that a Gag molecule is readily dissociated from a Gag-Syx6 pair during membrane fusion.

### Upregulation of TNFα secretion imposed by Syx6-mediated trafficking

HIV-1 infection is accompanied by dysregulation of cytokine production *in vivo* and *in vitro*, with downregulation of interleukin (IL)-2 and interferons and upregulation of proinflammatory cytokines IL-1, IL-4, IL-6, IL-10, and TNFα (94). The mechanism by which HIV-1 infection upregulates TNFα production involves HIV-1 accessory proteins, Nef, Vpr, and Tat, which activate the nuclear factor-kB signaling (95). We found that HIV-1-induced upregulation of TNFα secretion was dependent on Syx6 expression in our cell systems. The upregulation of TNFα secretion was moderated when HIV-1 lacked the MA domain, although both viruses induced TNFα expression to similar levels. Interestingly, TNFα and Gag were cotransported *via* Syx6-positive carriers (Fig. 7). These data suggest additional involvement in which Gag trafficking may facilitate TNFα secretion *via* Syx6. These hypotheses are supported by the findings that not only Gag but also TNFα can directly bind to Syx6 (Fig. 8). They bind to slightly discrete regions in Syx6: Gag interacts with the SNARE domain of Syx6 (Fig. 2), whereas TNFα interacts with the C-terminal region other than the SNARE (Fig. 8). However, it should be noted that they do not necessarily bind the same Syx6 molecule, but rather bind different Syx6 molecules on the same vesicle.

Unlike granulocytes, which stockpile cytokines in secretory granules, macrophages and T lymphocytes secret cytokines *via* the constitutive secretory pathways (72, 73, 96). In macrophages and T lymphocytes, TNFα is secreted by the transendosomal pathway through RE, which requires Syx7, Vti1b, and Syx6 (for TGN-to-RE trafficking) and subsequently Syx4 and SNAP23 (for RE-to-PM trafficking) (71, 72, 96). Interestingly, we found that the knockdown/knockout of SNAP23 in Jurkat cells severely reduced HIV-1 production (Fig. S6). It is possible that in immune cells, Gag may enter the transendosomal pathway mediated by Syx6 and SNAP23, where Gag and TNFα may overlap. Focusing on the SNAREs and pathways used for cytokine secretion may open new avenues for the study of transport pathways of viral components, which may explain the dysregulation of cytokine production in viral infection.

In summary, we provide evidence that Syx6 is a positive factor for Gag trafficking. Compared to the host factors previously reported to be responsible for Gag trafficking, the characteristic feature of Syx6 is that Syx6 is associated and cotransported with Gag during trafficking. Considering the model suggesting that Syx6 cycles between the TGN and the EE (38, 65), our observations suggest that Syx6 may play a role in delivering Gag to the next compartments and/or recycling Gag from the degradation pathways, although other possibilities cannot be ruled out.

## Experimental procedures

### Gene knockdown and knockout

Twenty-one nucleotide siRNA duplexes with symmetric 3′-dTT overhangs were purchased from B-Bridge (Cosmo Bio, Tokyo, Japan). The sense sequences and their targets were as follows: 5′-CAGCAUAGUUGAAGCAAAUTT-3′ (siSyx6-204, targeting nucleotide 204–222 of Syx6 ORF) and 5′-AGGCAUUAGCUGAAAGAAATT-3′ (siSyx6-335, targeting nucleotide 335–353 of Syx6 ORF), 5′-CAAGCAAGCUACAGGAAAATT-3′ (siSyx12–167, targeting nucleotide 167–185 of Syx12 ORF), and 5′-GCUCAGAGGUGCACGUCGATT-3′ (siSyx12–674, targeting nucleotide 674–692 of Syx12 ORF), 5′-CUACAACAGCCACAACGUCTT-3′ (targeting GFP, as control), and 5′-CGUACGCGGAAUACUUCGATT-3′ (targeting luciferase, as control). 80 nM siRNA was transfected using Lipofectamine2000 (Thermo Fisher Scientific, Waltham, MA) for each knockdown experiment.

For gene knockdown in immune cells, lentiviral particles expressing 3 of target-specific shRNAs (19–25 nt) and control shRNAs were purchased from Santa Cruz Biotechnology. For CRISPR-Cas9 systems, gRNAs (targeting nucleotides 408–427 and 553–572 of Syx6 ORF, selected using CRISPRdirect) were cloned into lenti-CRISPRv2 containing a Cas9 expression cassette (Addgene). Lenti-CRISPRv2 that did not contain gRNAs was used as control. 293T cells were cotransfected with lenti-CRISPRv2, psPAX2 (Addgene), and pHCMV-G (expressing VSV-G) to produce recombinant lentiviruses. Stably expressing cell lines were established with puromycin selection.

### DNA construction

For rescue of Syx6/Syx12 knockdown phenotypes, Syx6 containing siSyx6-335-mismatched sequence (AAGCCTTGGCCGAGAGGAA, mismatches underlined) and Syx12 containing siSyx12-167-mismatched sequence (CCAGTAAACTGCAAGAGAA, mismatches underlined) were cloned into a pCAGGS derivative in which the CMV immediate enhancer sequence was deleted.

The HIV-1 proviral clone pNL43 was used as the WT. A pNL43 derivative expressing Gag*Δ*MA was constructed by deleting the majority of the MA gene (nucleotide 821–1152), resulting in expressing the N-terminal 10 amino acids fused with the C-terminal 12 amino acids of MA. A pNL43 derivative with deletion of Env was constructed by deleting the nucleotide 6344 to 7250 in the *env* gene. Double amino acid substitutions in the membrane binding region of MA (K26E,K27E, K30E,K32E, and K39E,R43E) and in the H5 of MA (K103G,K110G, Q108A,A115G, and Q1116G,Q117G) were generated by PCR mutagenesis. pNL43 derivatives expressing Gag-EGFP and Gag-mSB were constructed by inserting the EGFP and mSB cDNAs to the end of the *gag* ORF with deletion the *pol* gene (nucleotide 2290–4553).

For immunoprecipitation, immunoisolation, and pulldown assays, the full-length of Syx6 cDNA (256 amino acids) and its fragments (N-terminal 116 amino acids, C-terminal 140 amino acids, and with deletion of SNARE domain 60 amino acids) were added by the Myc or FLAG tag sequence at their N-termini and were cloned into a mammalian expression plasmid pcDNA3. The full-length cDNAs encoding Syx16, Syx12, Vti1a, VAMP2, and VAMP4 were also added by the Myc or FLAG tag sequence at their N-termini and cloned into pcDNA3. For Gag expression, the full-length *gag* gene of HIV-1 (the HXB2 and NL strains) and its truncation fragments were tagged with FLAG or HA sequence at their C-termini and were cloned into mammalian expression plasmids pCI and pCAGGS. The Gag*Δ*MA, Gag mutants with double amino acid substitutions (described above), and additional Gag mutants with double amino acid substitutions in MA (R91G,K110G, D93G,K110G, K95G,K110G, K98G,K110G, E99G,K110G, D102G,K110G, E105G,K110G, E106G,K110G, E107G,K110G, K103G,E105G, K103G,E106G, K103G,E017G, K103G,K112G, K103G,K113G, and K103G,K114G) were generated by PCR mutagenesis and were tagged with FLAG sequence at their C-termini. The full-length of TNFα cDNA was added by the HA tag sequence at its N-terminus and cloned into pCAGGS. For GST pulldown, the *gag* truncation fragments were cloned into an *E. coli* expression plasmid pGEX-2T.

For confocal microscopy and live-cell imaging, the cDNAs encoding Gag-EGFP, Gag-mSB, and Gag-Cerulean were cloned into pCAGGS. The Gag*Δ*MA and Gag mutants with amino acid substitutions in MA were also fused with EGFP. Syx6 and Syx12 were fused with EGFP and mChr/mSB at the N-termini, as shown previously (37). AP1μ and AP3μ were fused with mSB at the C-termini. The Rab family proteins were fused with AcGFP at their N-termini and cloned into a derivative of pCAGGS. TNFα was fused with EGFP and Venus at the C-terminus (TNF-EGFP/Venus) and cloned into pCAGGS.

### HIV-1 production and replication

HeLa and 293T cells (American Type Culture Collection, Manassas, VA) were cotransfected with pNL43 and siRNA. Expression of HIV-1 proteins and host proteins was monitored by western blotting with mouse anti-HIV-1 p24CA (97), mouse anti-Syx6 (S55420, Transduction Laboratories), mouse anti-Syx12/13 (VAM-SV026, Stressgen), mouse anti-actin (Sigma-Aldrich) mAbs and human anti-HIV-1 gp120Env Ab (Immuno Diagnostics). HIV-1 particles were purified from the culture media by ultracentrifugation through 20% sucrose cushions. pNL43 derivatives (expressing Gag*Δ*MA and Gag with amino acid substitutions in MA) were similarly transfected to HeLa and 293T cells. The particle fractions were analyzed by western blotting with mouse anti-HV-1 p24CA mAb. The culture media were subjected to HIV-1 p24CA antigen capture ELISA (ZeptoMetrix, Buffalo, NY).

Jurkat and U937 cells (American Type Culture Collection) were transduced with lentiviruses expressing shRNAs and gRNAs. For HIV-1 replication, the transduced cells were infected with HIV-1 (the NL strain) at MOI of 0.1, and the culture medium was temporally collected (twice a week). For HIV-1 production, the cells were infected with at MOI of 3. HIV-1 yields were quantified by p24CA antigen capture ELISA.

Yeast genetic mutants, whose defects impact post-Golgi membrane trafficking (Table S1), were transformed with yeast expression plasmid pKT10 containing the HIV-1 *gag* gene. Following the removal of the cell wall, yeast spheroplasts were cultured overnight to produce Gag particles to the culture media, as described previously (44). They were also transformed with Gag-EGFP expression plasmid.

### TNFα production

Jurkat, U937, and THP-1 cells (American Type Culture Collection) were treated with 100 ng/ml PMA for differentiation (74, 75) and infected with HIV-1 (the NL strain) at MOI of 2. At 2 days postinfection, the culture medium was collected and subjected to quantification of TNFα release by ELISA (DTA00D, R&D Systems). In some experiments, cells were lysed with 0.5% Triton X-100 and similarly subjected to ELISA.

### Coimmunoprecipitation and pulldown

293T cells were cotransfected with various combinations of the constructs expressing the tag sequence-added Gags, Syx6, Syx16, Syx12, VAMP2, VAMP4, and TNFα and lysed in lysis buffer (50 mM HEPES [pH 7.4], 150 mM NaCl, 1.5 mM MgCl_2_, 1 mM EGTA, 1% Triton-X 100, 10% Glycerol, 1 mM DTT, protease inhibitors) for 30 min. After clarification by centrifugation at 15,000*g* for 30 min, the supernatants were incubated with mouse anti-FLAG (Sigma-Aldrich) or anti-HA mAb (Sigma-Aldrich) and subsequently with protein G-sepharose 4 FF beads (Pharmacia, Cytiva). In some experiments, anti-FLAG mAb-immobilized agarose beads (Sigma-Aldrich) were used. The immunoprecipitates were analyzed by western blotting with mouse anti-FLAG, anti-Myc (Abcam), and anti-HA mAbs.

For GST pulldown, the *gag* fragments of HIV-1 (the HXB2 strain) were cloned into pGEX-2T and expressed in *E. coli*. The GST-fused Gag fragments were purified (98) using glutathione-sepharose 4 FF beads (Pharmacia, Cytiva). [^35^S]-labeled Syx6 was produced by using *in vitro* transcription/translation coupled system, according to the manufacturer’s instructions (Promega, Madison, WI) and incubated with the glutathione-sepharose beads capturing purified GST-Gag fragments. FLAG/HA sequence-tagged Gag, Syx6, and TNFα were expressed in 293T cells and lysed with lysis buffer. FLAG pulldown was performed with anti-FLAG mAb-immobilized agarose beads. After immobilized onto the beads, they were incubated with cell lysates expressing HA-tagged targets (prey). HA pulldown was with anti-HA mAb and protein G-sepharose beads and the precipitates were incubated with FLAG-tagged targets (prey).

### Immunoisolation of the membrane/vesicle fractions

293T cells were cotransfected with various combinations of the constructs of the tag sequence-added Gag, Syx6, and TNFα. Following brief sonication, the cell lysates were adjusted to 70% (wt/vol) sucrose, layered by 65% and 10% (wt/vol) sucrose step gradients, and subjected to equilibrium flotation centrifugation overnight at 4 °C (52). The membrane-bound fraction was subjected to immunoisolation with mouse anti-FLAG and anti-HA mAbs and the precipitates were analyzed by western blotting.

### Confocal microscopy and electron microscopy

HeLa and 293T cells were cotransfected with various combinations of EGFP/AcGFP-fused antigen and mSB/mChr-fused antigen expression plasmids: Gag-EGFP and mSB/mChr-Syx6; Gag-mSB and TNF-EGFP; AcGFP-Rab and Gag-mSB or mSB-Syx6. The cells were fixed with 3.7% paraformaldehyde and subsequently with chilled methanol and subjected to confocal microscopy (Leica, Wetzlar, Germany). Triple expression was performed by cotransfection with Gag-Cerulean, AcGFP-Rab or TNF-Venus, and mChr-Syx6 expression plasmids. In some experiments, pNL43 derivatives expressing Gag-EGFP and Gag-mSB were used instead of Gag-EGFP and Gag-mSB expression plasmids. For disruption of microtubules, nocodazole was added at 10 μg/ml at 4 h posttransfection. Pearson’s correlation coefficients (in more than 3 cells) were calculated using coloc2 in ImageJ/Fiji. For endogenous Syx6/Syx12, cells were immunostained with mouse anti-Syx6 (S55420, Transduction Laboratories) and anti-Syx12/13 (VAM-SV026, Stressgen) mAbs. In some experiments, cells were costained with mouse anti-CD63 mAb (KILL150A, Santa Cruz Biotechnology). Nuclei were stained with DAPI.

Jurkat cells transduced with lentiviruses expressing shRNAs and gRNAs were infected with HIV-1 (NL strain) at MOI of 5. At 2 days postinfection, the cells were placed on 0.1% poly-L-lysine-coated coverslips, fixed, and immunostained with anti-HIV-1 p17MA mAbs specific for the mature p17MA C-terminal epitope (ARP316 or 342, Centre for AIDS Reagents, NIBSC, UK) and FITC-labeled anti-HIV-1 p24CA mAb (1103-F, Immuno Diagnostics, Woburn, MA). Nuclei were stained with DAPI.

For TNFα expression, U937 cells were transduced with lentiviruses expressing shRNAs and gRNAs. U937 and THP-1 cells were treated with 100 ng/ml PMA and infected with HIV-1 (the NL strain) at MOI of 2. At 2 days postinfection, the cells were placed on poly-L-lysine-coated coverslips and fixed with 3.7% paraformaldehyde and chilled methanol. The cells were immunostained with anti-TNFα rabbit Ab (GTX110520, GeneTex) and anti-HIV-1 p24CA mAb. Nuclei were stained with DAPI.

For electron microscopy, HeLa and 293T cells were transfected with siRNA and subsequently with pNL43. At 2 days posttransfection with pNL43, the cells were fixed with 2% glutaraldehyde in 100 mM cacodylate buffer (pH 7.2) and postfixed with 1% osmium tetroxide. For immunoelectron microscopy, yeast spheroplasts were fixed in 0.5% glutaraldehyde in PBS and sections were incubated with anti-HIV-1 p24CA mAb coupled with colloid gold.

### Live-cell imaging

HeLa cells (in glass bottom dishes) were cotransfected with Gag-EGFP and mChr-Syx6 or mChr-Syx12 expression plasmids. In some experiments, HeLa cells were cotransfected with Gag-EGFP and mSB-Syx6 expression plasmids and siRNA. For disruption of microtubules, cells were treated with 10 μg/ml. For microtubule-dependent trafficking, EGFP-Syx6 and Gag-EGFP expression constructs were cotransfected with mSB-tubulin expression plasmid. For TNFα expression, Gag-mSB and mSB-Syx6 were coexpressed with TNF-EGFP in HeLa cells. The cells were incubated at 37 °C in a microscope stage top incubation chamber. Live-cell imaging was performed at 20 to 24 h posttransfection with a confocal microscope (IX71, Olympus Optical, Japan) equipped with a microlens-enhanced Nipkow-disk confocal scanner unit (CSUX1, Yokogawa Electric, Japan). Sequential Images (excitation at 488 and 568 nm) were acquired at 1-s intervals for 100 s with an electron-multiplying CCD camera (Luca, Andor Technology). Bleach and contrast corrections of images were performed using ImageJ software, and tracking of punctate fluorescent signals using MTrackJ plugin created by Eric Meijering (http://www.imagescience.org/meijering/software/mtrackj).

### Statistical analysis

All the results were the representatives of two or three independent experiments. The data in line and bar graphs were presented as the mean with SD. Statistical analysis was performed using the Mann–Whiteny *U* test, where *p* < 0.05 was considered statistically significant.

## Data availability

All data are contained with the manuscript.

## Supporting information

This article contains supporting information (99, 100, 101, 102, 103, 104, 105, 106, 107, 108, 109).

## Conflict of interest

The authors declare that they have no known competing financial interests or personal relationships that could have appeared to influence the work reported in this paper.
