# Supplementary material for: Intracellular trafficking of HIV-1 Gag via Syntaxin 6-positive compartments/vesicles: Involvement in tumor necrosis factor secretion
Source: J Biol Chem. 2024 Jan 26;300(3):105687. doi: 10.1016/j.jbc.2024.105687 (PMC10891346; doi:10.1016/j.jbc.2024.105687)
Supplement: Supporting Information [file mmc6.docx]

Title:

**Intracellular trafficking of HIV-1 Gag via Syntaxin 6-positive compartments/vesicles: involvement in tumor necrosis factor secretion**

Authors:

Naomi Tsurutani^1,#a^, Fumitaka Momose^1,#b^, Keiji Ogawa^1,#c^, Kouichi Sano^2^, and Yuko Morikawa^1,^*

**Supporting information**

A list of the materials included:

Text S1. A SNARE molecule Tlg1 is responsible for HIV-1 Gag particle release in yeast.

Table S1. Yeast genetic mutants used in this study.

Figure S1. HIV-1 Gag localization and Gag particle release in yeast genetic mutants.

Figure S2. Knockdown phenotypes of Syx6/Syx12 and rescue of their phenotypes by siRNA-resistant Syx6/Syx12 expression.

Figure S3. Gag partially colocalize with Syx6 and Syx12 in 293T cells and their knockdown impairs Gag localization to the PM and particle production.

Figure S4. HIV-1 Gag localization in coexpression of Gag-EGFP with the WT Gag and in single expression of Gag-EGFP.

Figure S5. The charged amino acids in the H5 of Gag MA are responsible for the interaction with Syx6.

Figure S6. Knockdown and knockout of SNAP23 severely impair HIV-1 replication in Jurkat cells.

Movie S1. HIV-1 Gag is transported via Syx6-positive compartments/vesicles in an MA-dependent fashion.

Movie S2. Gag and Syx6 are partly transported along microtubules.

Movie S3. Live-cell imaging of Gag MA mutants with mChr-Syx6.

Movie S4. TNFα is cotransported with Gag and Syx6.

Movie S5. Gag is not cotransported with AP1 or AP3.

**Text S1.**

**A SNARE molecule Tlg1 was responsible for HIV-1 Gag particle release in yeast.**

Yeast genetic mutants we used are defective in post-Golgi membrane trafficking: deletions of the SNARE and endosomal vacuolar protein sorting (vps) proteins (Table S1). The SNARE mutants involve EE, LE/prevacuolar, and vacuolar defects, respectively. The vps mutants are largely classified in two groups: retrograde (endosome-to-Golgi) transport defect (in class A) and defects in the ESCRT machinery (in class E). Following expression of HIV-1 Gag protein, Gag particles were produced from the yeast spheroplasts, as described previously (44). Broadly equivalent levels of Gag protein were expressed in all genetic mutants but that the levels of Gag particles production varied (Fig. S1A): In SNARE-deleted group, *tlg1∆* cells, which are defective in transport between the late Golgi and EE, displayed severe reduction in Gag particle release (to 10-15%). *pep12∆* cells, which are defective in transport from the late Golgi to LE/prevacuole, showed a modest reduction in Gag particle release. In contrast, none of *vps∆* cells tested showed a significant reduction in Gag particle release. The class E vps molecules (ESCRTs), essential for HIV-1 particle budding in human cells (10-12), were not required for Gag particle release in yeast, confirming previous studies in which Gag particle budding was neither ESCRT- nor L domain-dependent in yeast (44, 99). We find that Gag does not efficiently bind VPS23, yeast orthologue of human TSG101 in coimmunoprecipitation and yeast two-hybrid assays (data not shown). These findings suggest that the yeast cell system is not functionally equivalent of mammalian cells at the stage of viral particle budding and contains alternative machinery for particle budding. Our study also used retrograde (endosome-to-Golgi) transport mutants because HIV-1 Gag is frequently distributed in endosomes; however, no significant reduction in particle production was observed.

The yeast genetic mutants were similarly transformed with Gag-EGFP expression plasmid and subjected to confocal microscopy (Fig. S1B). Gag-EGFP was targeted to and accumulated at the PM in the WT cells. All the tested *vps∆* cells exhibited Gag targeting to the PM. In *tlg1∆* cells, Gag-EGFP rarely accumulated at the PM but diffusely distributed in the cytoplasm. Punctate accumulation of Gag-EGFP in the cytoplasm was prominent in *pep12∆* cells where it was frequently accompanied by a diffuse distribution of Gag-EGFP in the cytoplasm. These subcellular localizations were confirmed by immunoelectron microscopy (Fig. S1C). These results indicate that in particle production-impaired cells, a Gag fraction is missorted to intracellular membranous compartments.

**Table S1. Yeast genetic mutants used in this study.**

Genotype Defect Reference Human

orthologue

wild type (100)

SNARE defects

*tlg1∆* fusion of EE with late Golgi (101) Syx6

*pep12∆* fusion with LE/prevacuole (102) Syx12

*vam3∆* fusion with vacuole (103) Syx7

VPS defects

*vps27∆* an ESCRT-0 molecule for MVB pathway (104) HRS

*vps23∆* an ESCRT-I molecule for MVB pathway (105) TSG101

*vps4∆* ATPase for vacuolar transport (106) VPS4

*vps45∆* a Sec1p/Munc18 (107) VPS45

*vps10/pep1∆* vacuolar protein sorting receptor (108) VPS10

*vps29/pep11∆* an endosome-to-Golgi retromer (109) VPS29

*vps35∆* an endosome-to-Golgi retromer (109) VPS35

**
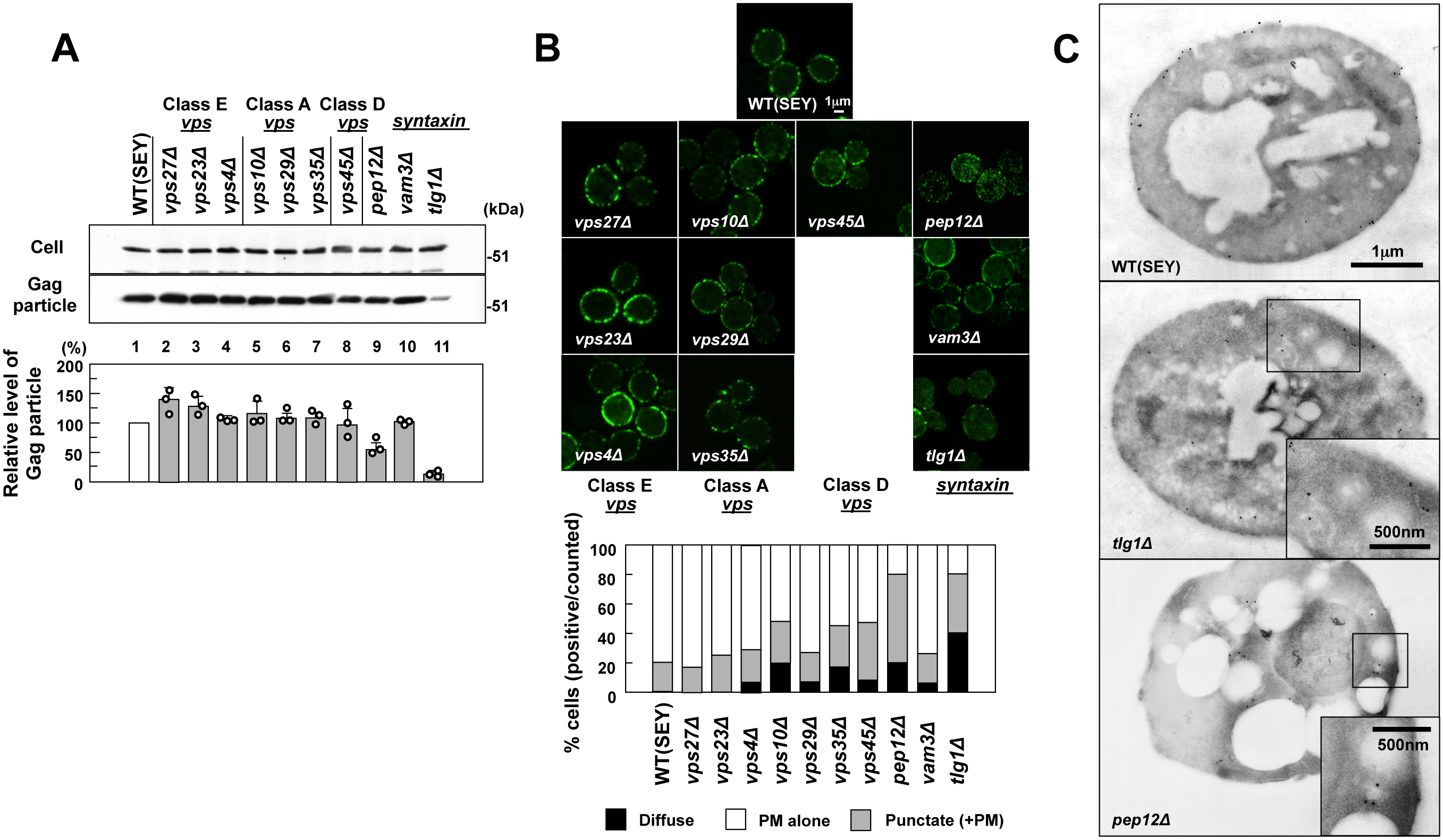
**

**Figure S1. HIV-1 Gag localization and Gag particle release in yeast genetic mutants.** *A*, Gag particle release in yeast genetic mutants. Yeast genetic mutants were transformed with a yeast expression plasmid pKT10 containing the full-length of HIV-1 *gag* gene. Yeast transformants were grown at 25˚C in synthetic defined medium (0.67% yeast nitrogen base, 2% glucose, and amino acid mixtures without uracil or tryptophan) to OD_600_=1.5-2.0. Spheroplast formation was performed by Zymolyase-T100 digestion and the spheroplasts were maintained under isotonic conditions for 2 h. For purification of Gag particles, the culture media were subjected to ultracentrifugation with 20-70% (wt/vol) sucrose gradients overnight. The cells (0.5 OD units) and Gag particle fractions (equivalent to 10 OD units of cells) were analyzed by western blotting with anti-HIV-1 p24CA mAb. Representative blots are shown. Data were semi-quantified using ImageJ software and the mean levels of Gag particles in the WT cells (SEY) were set at 100%. *B*, Intracellular localization of Gag-EGFP in yeast genetic mutants. Yeast genetic mutants were transformed with pKT10 containing HIV-1 *gag-egfp* gene. Scale bar = 1 µm. 100-150 EGFP-positive cells were subjected to confocal analysis for Gag-EGFP distribution patterns (diffuse in the cytoplasm, at the PM alone, or cytoplasmic puncta). The numbers of cells with each pattern of Gag-EGFP distribution were counted. *C*, Immunoelectron microscopy. Sections of yeast spheroplasts expressing Gag were stained with anti-p24CA mAb coupled with gold. Scale bars: 1 µm (panels); 500 nm (insets).


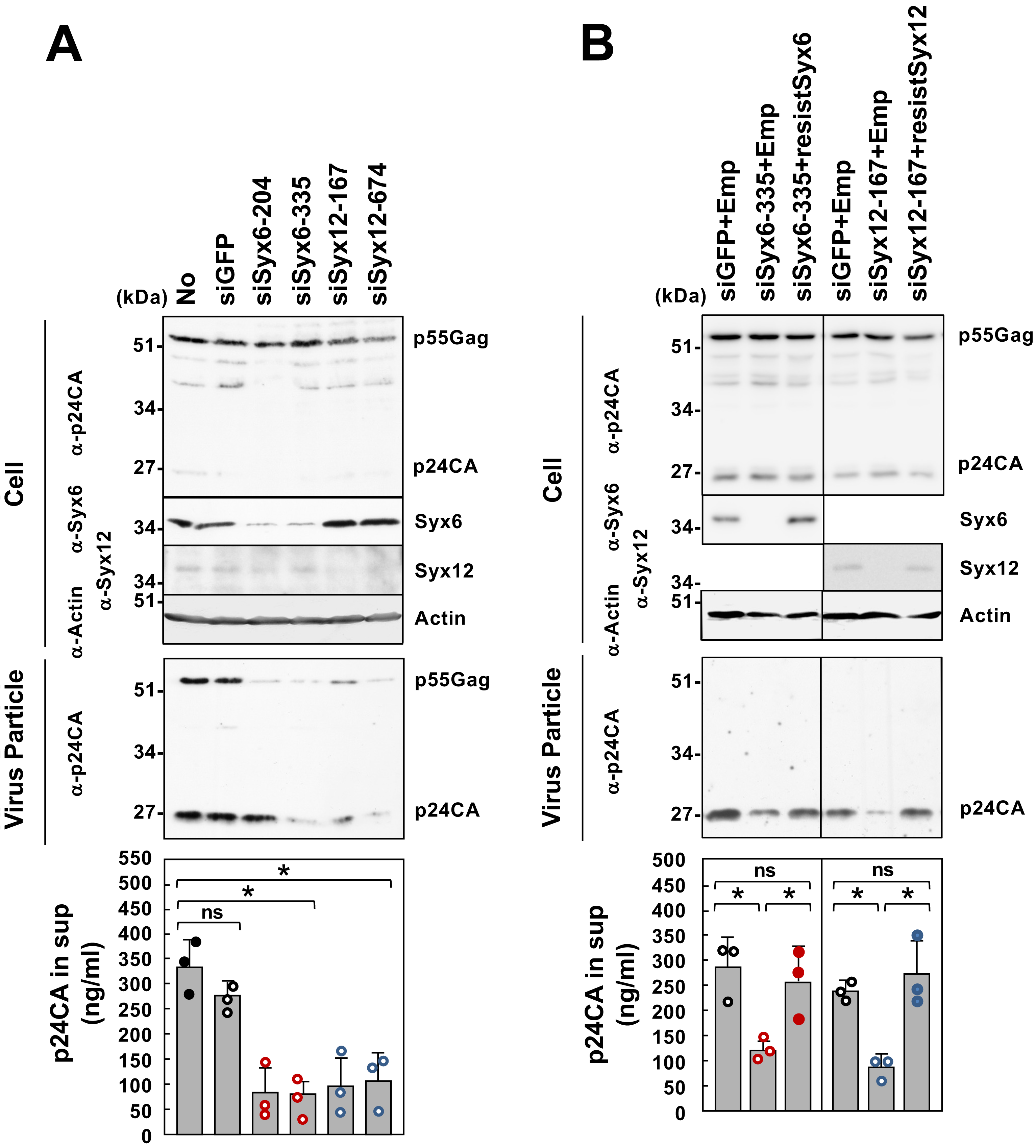


**Figure** **S2. Knockdown phenotypes of Syx6/Syx12 and rescue of their phenotypes by siRNA-resistant Syx6/Syx12 expression.** *A*, Knockdown of Syx6 and Syx12 impairs particle release of Env-deficient HIV-1. 293T cells (in 6-cm dishes) were transfected with 80 nM Syx6 siRNAs (siSyx6-204 and siSyx6-335), Syx12 siRNAs (siSyx12-167 and siSyx12-674), or GFP siRNA (siGFP, control) and then 1 µg of a pNL43 derivative with deletion of the *env* gene. At 48 h posttransfection, 293T cells and purified HIV-1 particles were analyzed by western blotting using anti-HIV-1 p24CA, anti-Syx6, anti-Syx12, and anti-actin mAbs. *B*, Rescue of Syx6/Syx12 knockdown phenotypes. 293T cells (in a 6-well plate) were cotransfected with 80 nM siRNA (Syx6-335, siSyx12-167, or siGFP) and a pCAGGS derivative expressing siSyx6-335-resistant Syx6 (0.2 µg) or siSyx12-167-resistant Syx12 (0.04 µg). After 48 h incubation, the cells were reseeded and transfected with 0.5 µg of pNL43. At 48 h posttransfection with pNL, the cells were similarly analyzed by western blotting. Representative blots are shown. Particle yields were quantified by HIV-1 p24CA antigen ELISA. Data are the mean with SD from 4 independent experiments. *, *p* < 0.05; ns, not significant, Mann-Whiteny U test.

**
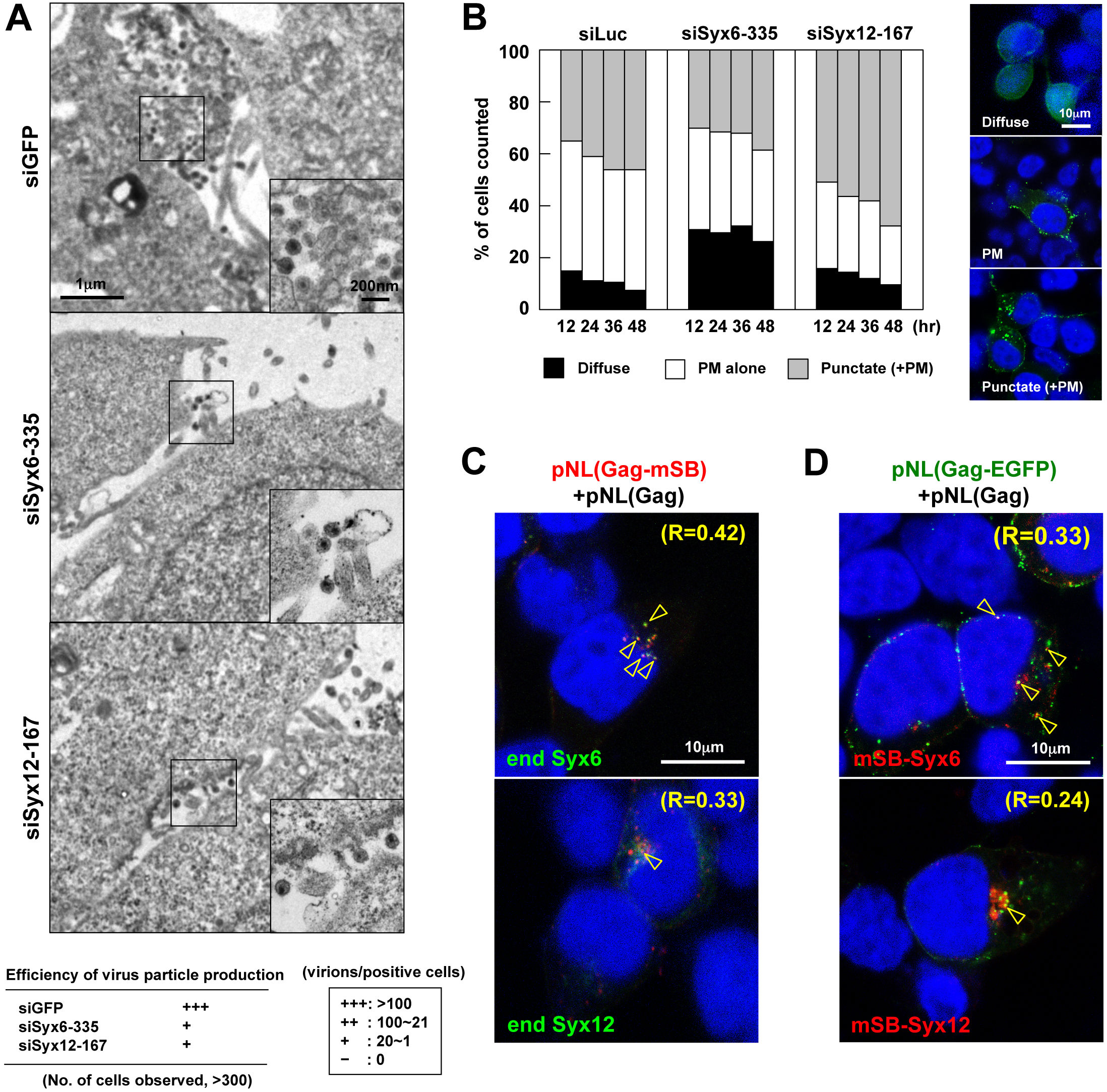
**

**Figure S3. Gag partially colocalize with Syx6 and Syx12 in 293T cells and their knockdown impairs Gag localization to the PM and particle production.** *A and B*, Knockdown of Syx6 and Syx12. 293T cells (in 12-well plates) were transfected with 80 nM siSyx6-335, siSyx12-167, or siGFP and with 0.2 µg of pNL43 (*A*), or 80 nM siSyx6-335, siSyx12-167, or siLuc and with 0.2 µg of a pNL derivative expressing Gag-EGFP (*B*). *A*, Electron microscopy. Panels: upper, siGFP-transfected; middle, siSyx6-335-transfected; lower, siSyx12-167-transfected. Scale bars: 1 µm (panels); 200 nm (insets). *B*, Intracellular localization of Gag. At several time points, cells were fixed and subjected to confocal microscopy. Nuclei were stained with DAPI. Representative images of Gag localization (diffuse in the cytoplasm, at the PM alone, or cytoplasmic puncta) were shown. Gag-EGFP-positive cells at each time point (n = 150-200) were subjected to quantification of Gag localization. *C and D*, Colocalization of Gag with Syx6. *C*, 293T cells were transfected with a pNL derivative expressing Gag-mSB and immunostained for endogeneous Syx6 or Syx12 with anti-Syx6 or anti-Syx12/13 mAbs. *D*, 293T cells were cotransfected with a pNL derivative expressing Gag-EGFP expression plasmid and mSB-Syx6 or mSB-Syx12 expression plasmid (at a Gag-to-Syx DNA ratio of 4:1). Representative confocal images of 24 h postransfection were shown. *Yellow arrowheads* indicate colocalization of antigens. R indicates Pearson’s correlation coefficient of the antigens.

**
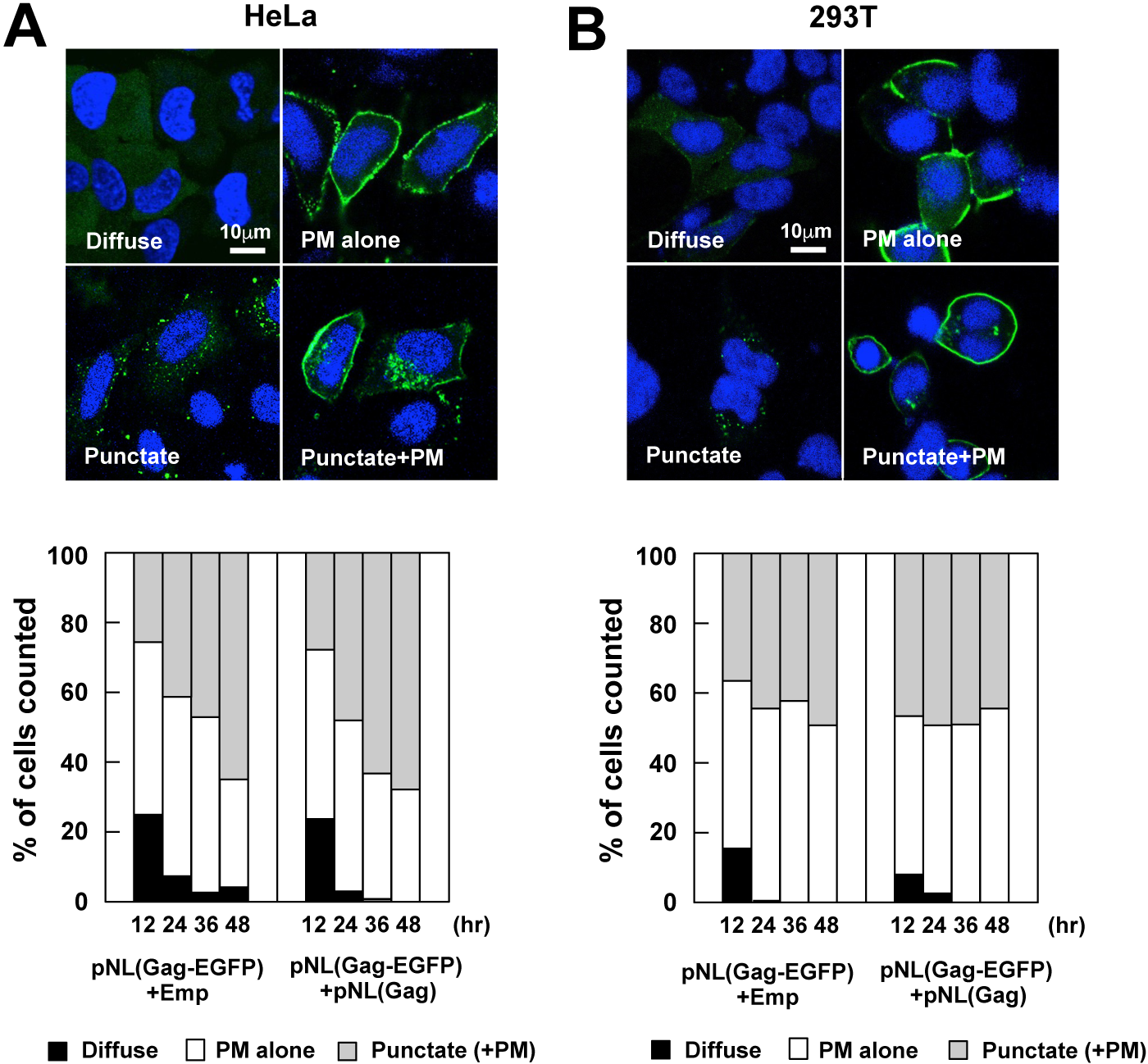
**

**Figure S4. HIV-1 Gag localization in coexpression of Gag-EGFP with the WT Gag and in single expression of Gag-EGFP.** HeLa (*A*) and 293T (*B*) cells were cotransfected with a pNL derivative expressing Gag-EGFP and a pNL derivative expressing untagged WT Gag (with deletion the *pol* gene). Cells were also singly transfected with a pNL derivative expressing Gag-EGFP. The cells were fixed at several time points and subjected to confocal microscopy. Nuclei were stained with DAPI. For quantification of Gag localization, Gag-EGFP-positive cells (n = 90-150) were observed at each time point and the number of cells with each pattern of Gag distribution (diffuse in the cytoplasm, at the PM alone, or cytoplasmic puncta) was counted.

**
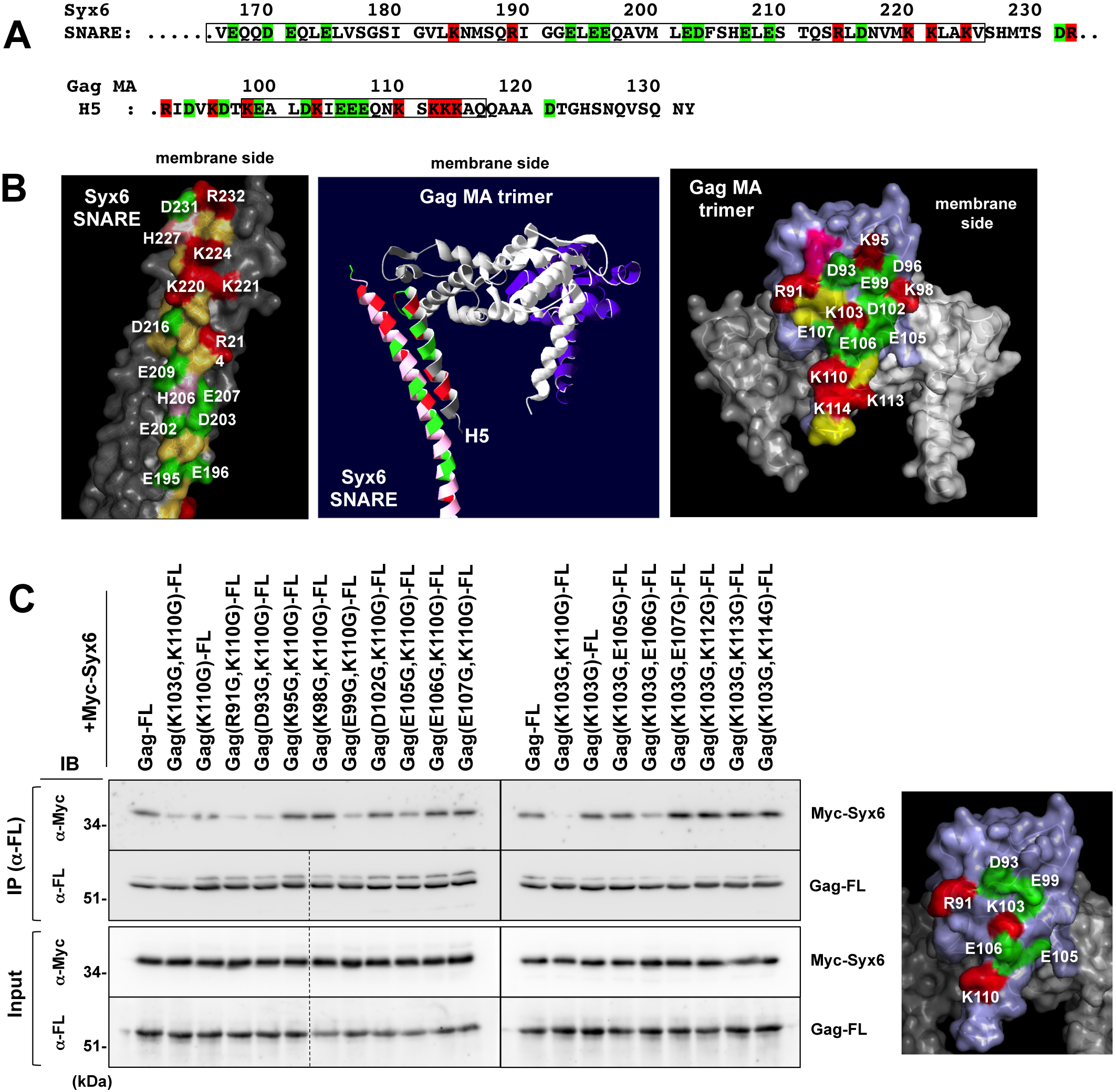
**

**Figure S5. The charged amino acids in the H5 of Gag MA are responsible for the interaction with Syx6.** *A*, The amino acid sequences of Syx6 SNARE and MA H5. *B*, The protein surface models of Syx6 SNARE (*left*) and Gag MA trimer (*right*) were generated from PDB accession numbers 2NPS (DOI: 10.2210/pdb2NPS/pdb) and 1HIW, respectively, with PyMOL. Negatively charged amino acids are shown in green; positively charged amino acids in red; others in yellow. The interaction model of Gag MA H5 and Syx6 SNARE was shown in ribbon diagram (*middle*). *C*, The Gag MA mutant-Syx6 interaction. Dash lines indicate that one lane (another Gag mutant) was excised from the original blots. The amino acids that show a marked reduction in coimmunoprecipitation by substitutions are shown in the PyMOL model of Gag MA (*right*).

**
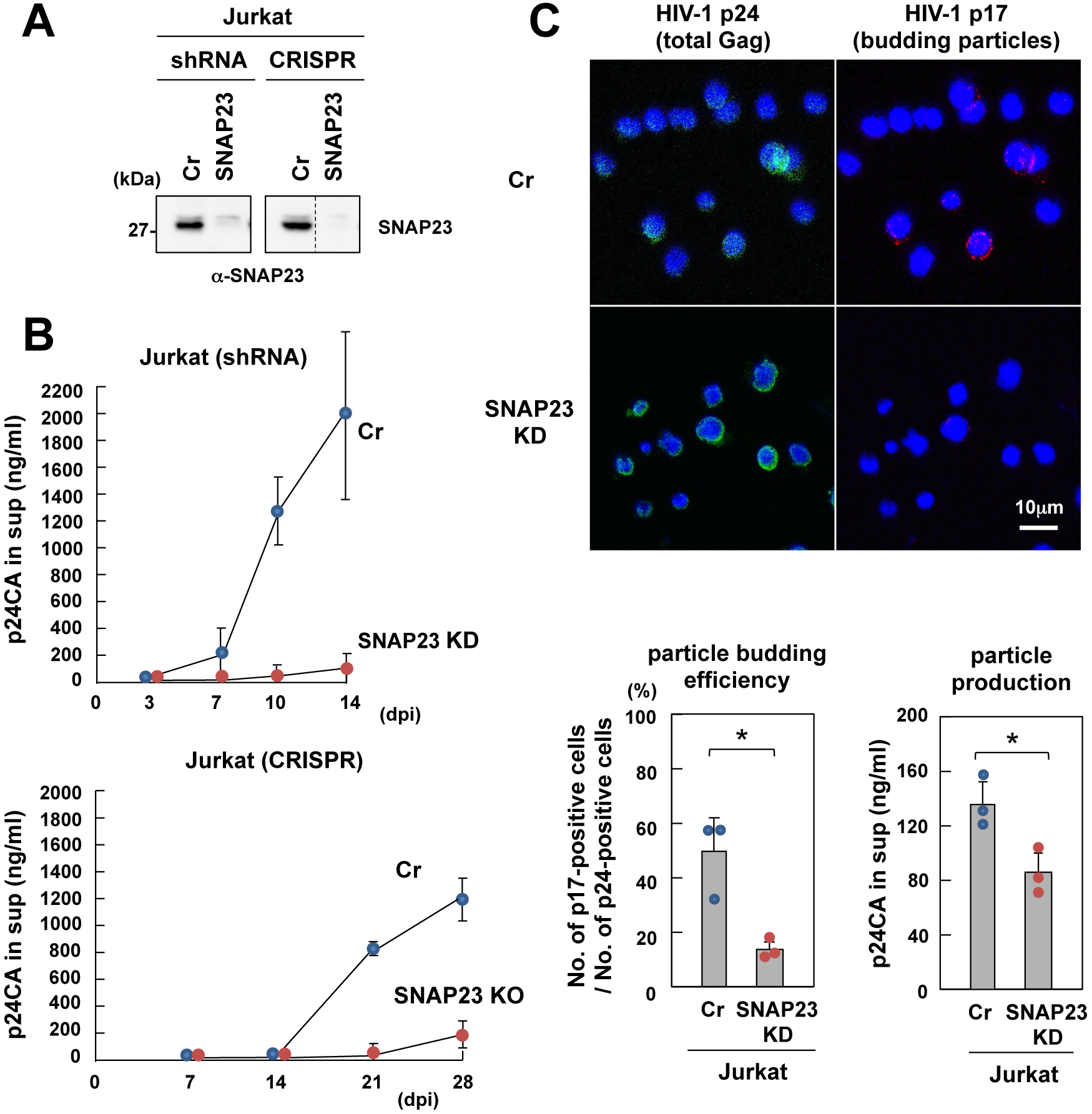
**

**Figure S6. Knockdown and knockout of SNAP23 severely impair HIV-1 replication in Jurkat cells.** *A*, Jurkat transduced with lentiviruses expressing 3 distinct SNAP23 shRNAs or scramble control shRNAs (Santa Cruz Biotechnology) were subjected to western blotting with rabbit anti-SNAP23 Ab (Synaptic Systems, Göttingen, Germany). Cells transduced with lentivirus expressing SNAP23 gRNA and Cas9 were cloned and then analyzed. Cells transduced lentivirus expressing Cas9 alone were used as control. A dash line indicates that one lane (the SNAP23 gRNA-transduced cell mixture) was excised from the original blot. *B*, HIV-1 replication. The SNAP23-knockdown/knockout Jurkat cells were infected with HIV-1 at MOI of 0.1. HIV-1 particle yields were temporally quantified by HIV-1 p24CA antigen ELISA. Data are the mean from 2 independent experiments. *C*, HIV-1 particle release from Syx6-knockdown Jurkat cells. SNAP23-knockdown Jurkat cells and scramble control cells were infected with HIV-1 at MOI of 5. At 48 h postinfection, the cells were immunostained with anti-HIV-1 p17MA mAb followed by Alexa Fluor 568-conjugated anti-mouse IgG and costained with FITC-labeled anti-HIV-1 p24CA mAb. More than 350 cells were subjected to analysis in each group. The efficiency of particle budding was assessed the ratio of the number of p17MA-positive cells to that of p24CA-positive cells (*lower left*). Levels of p24CA antigens in culture media were quantified by p24CA capture ELISA (*lower right*). The mean with SD from 3 independent experiments. *, *p* < 0.05, ns, not significant, Mann-Whiteny U test.

**Movie S1. HIV-1 Gag is transported via Syx6-positive compartments/vesicles in an MA-dependent fashion.** HeLa cells (in a glass bottom dish) were cotransfected with the following combinations of expression plasmids for: Gag-EGFP and mChr-Syx6 (*upper left*); Gag-EGFP, untagged Gag, and mChr-Syx6 (*upper middle*); Gag∆MA and mChr-Syx6 (*upper left*); Gag-EGFP and mChr-Syx6 plus 80 nM luciferase siRNA (siLuc, control) (*lower left*); Gag-EGFP and mChr-Syx6 plus 80 nM Syx6 siRNA (siSyx6-335) (*lower middle*); Gag-EGFP and mChr-Syx12 (*lower right*). At 20-24 h posttransfection, cells were subjected to live-cell imaging. Dual color images were sequentially acquired at 1-s intervals. Movie (mp4 container format) was compressed at 10 frames/s, using H.265 (codec).

**Movie S2. Gag and Syx6 are transported along microtubules.** HeLa cells (in a glass bottom dish) were cotransfected with expression plasmids for mSB-tubulin and EGFP-Syx6 (*left*) or Gag-EGFP (*middle*). HeLa cells were cotransfected with expression plasmids for mChr-Syx6 and Gag-EGFP and treated with 10 µg/ml Nocodazole (*right*). Live-cell imaging was performed as described in the legend for Movie S1. Movie (mp4 container format) was compressed at 10 frames/s, using H.265 (codec).

**Movie S3. Live-cell imaging of Gag MA mutants with mChr-Syx6.** HeLa cells (in a glass bottom dish) were cotransfected with expression plasmids for mChr-Syx6 and Gag MA mutants fused with EGFP. The following Gag MA point mutants were used: (K26E,K27E), (K30E,K32E), (R39E,R42E), (K103G,K110G), (Q108G,A115G), and (Q116G,Q117G). Live-cell imaging was performed as described in the legend for Movie S1. For comparison, the movie for coexpression of WT Gag-EGFP with mChr-Syx6 (shown in S1 Movie) was also shown. Movie (mp4 container format) was compressed at 10 frames/s, using H.265 (codec).

**Movie S4. TNFα is cotransported with Gag and Syx6.** HeLa cells were cotransfected with expression plasmids for TNFα-EGFP and Gag-mSB (*left*) or mSB-Syx6 (*right*). Live-cell imaging was performed as described in the legend for Movie S1. Movie (mp4 container format) was compressed at 10 frames/s, using H.265 (codec).

**Movie S5. Gag is not cotransported with AP1 or AP3.** HeLa cells were cotransfected with expression plasmids for Gag-EGFP and AP1µ-mSB (*left*) or AP3µ-mSB (*right*). Live-cell imaging was performed as described in the legend for Movie S1. Movie (mp4 container format) was compressed at 10 frames/s, using H.265 (codec).
